# Supplementary material for: Machine learning-guided co-optimization of fitness and diversity facilitates combinatorial library design in enzyme engineering
Source: Nat Commun. 2024 Jul 29;15:6392. doi: 10.1038/s41467-024-50698-y (PMC11289365; doi:10.1038/s41467-024-50698-y)
Supplement: Supplementary file 1 — Supplementary Information [file 41467_2024_50698_MOESM1_ESM.pdf]

Machine learning-guided co-optimization of fitness and diversity facilitates combinatorial library design in enzyme engineering

Kerr Ding[1,†], Michael Chin[2,†], Yunlong Zhao[2,†], Wei Huang[2], Binh Khanh Mai[3], Huanan Wang[2], Peng Liu[3,*], Yang Yang[2,4,*], Yunan Luo[1,*]

[1] School of Computational Science and Engineering, Georgia Institute of Technology, Atlanta, Georgia 30332, USA

[2] Department of Chemistry and Biochemistry, University of California, Santa Barbara, California 93106, USA

[3] Department of Chemistry, University of Pittsburgh, Pittsburgh, Pennsylvania 15260, USA

[4] Biomolecular Science and Engineering (BMSE) Program, University of California, Santa Barbara, California 93106, USA

[†] These authors contributed equally.

[*] E-mail: pengliu@pitt.edu; yang@chem.ucsb.edu; yunan@gatech.edu

Contents

A Supplementary Information **1**

A.1 Benchmarking datasets for computational experiments 1

A.2 Co-optimization of the fitness and diversity of the library 2

A.3 Zero-shot protein fitness prediction 4

A.4 Structure-based filter 5

A.5 High-quality starting library design for GB1 6

A.6 High-quality starting library design for CreiLOV 8

A.7 Experimental validation of MODIFY on engineering cytochrome *c* 9

A.8 Classical molecular dynamics (MD) simulations. 13

B Supplementary Figures **15**

C Supplementary Tables **23**

A Supplementary Information

A.1 Benchmarking datasets for computational experiments

In our work, we evaluated MODIFY for zero-shot protein fitness prediction and starting library design on multiple benchmarking datasets curated by previous works.

ProteinGym. ProteinGym[1] is a benchmark dataset with 87 Deep Mutational Scanning (DMS) studies, which covers a wide range of protein families and also fitnesses (e.g., ligand binding and thermostability). We collected all single mutations from the 87 DMS studies and used the experimental data to evaluate the zero-shot ensemble approach in MODIFY for robust mutation effects prediction across diverse proteins. As three of the five models integrated in MODIFY (EVmutation, EVE, and MSA Transformer) by default were not trained on the low-coverage columns of MSA (i.e., column coverage lower than 70%) (Supplementary Information A.3), we only evaluated MODIFY on mutants whose mutation sites are in columns with coverage no less than 70%. ProteinGym stratified the 87 DMS studies based on the MSA depth of their target proteins[1]. The MSA depth is defined as N_{eff}/L, where L is the length covered, and N_{eff} refers to the effective number of sequences in the MSA[2]. In specific, proteins with $N_{\text{eff}}/L < 1$ have low MSA depth; proteins with $1 < N_{\text{eff}}/L < 100$ have medium MSA depth; proteins with $N_{\text{eff}}/L > 100$ have high MSA depth. Intuitively, proteins with lower MSA depth have fewer homologous sequences and are deemed more challenging than proteins with higher MSA depth for mutation effects prediction. For formatting purposes, we used abbreviations for the DMS dataset names in the ProteinGym substitution benchmark dataset shown in Fig. 2. We provided the mapping from the abbreviations to the DMS dataset names in Supplementary Table 1. ProteinGym v1.0 benchmark dataset[3] is a recently released extension of the ProteinGym benchmark dataset, which contains 217 DMS assays. The 217 DMS assays are categorized into five different function types: catalytic and biochemical activity, binding, expression, organismal fitness, and stability. We provided the mapping from the abbreviations to the DMS dataset names in Supplementary Table 2.

High-order GB1 mutants dataset. The fitness landscape of GB1 at sites 39, 40, 41, and 54 was systematically determined through experiments by Wu et al.[4]. Among the total $20^4 = 160,000$ variants, 149,361 variants have reliable experimental fitness values, and the fitness of the remaining variants was imputed through regularized regression. For zero-shot prediction performances, we solely evaluated MODIFY on variants with experimentally determined fitness. When assessing MODIFY for starting library design, we additionally included the variants with imputed fitness (10,639 variants). The fitness of the variants of GB1 is characterized by both stability (fraction of folded proteins) and function (binding affinity to IgG-Fc). The fitness of the wild-type protein (WT) is set as 1.0. For each variant, its fitness value is computed as relative to the WT. A mutant with a fitness value higher than 1.0 is considered beneficial, whereas a mutant with a fitness value lower than 1.0 is considered inferior to the WT. The lowest possible fitness value is 0.0.

High-order CreiLOV mutants dataset. Chen et al.[5] experimentally characterized a combinatorial mutagenesis library on CreiLOV across 15 sites (3, 4, 5, 7, 29, 34, 47, 60, 61, 92, 96, 98, 107, 109, and 113). CreiLOV is a prototype flavin mononucleotide (FMN)-based fluorescent protein (FbFP) from *Chlamydomonas reinhardtii*. Due to their oxygen-independent fluorescence, FbFPs are recognized as potential alternatives to the green fluorescent protein (GFP)[6]. Different from the landscape of GB1, this combinatorial library only spans 20 single mutations, which were previously determined to be beneficial or neutral through singe-site saturation mutagenesis. The

fluorescence value is used to represent the fitness of CreiLOV variants. A higher fluorescence value would indicate a better fitness for the given variant. Out of the 184,320 mutants, 165,428 of them had reliable experimental fitness values. For both library design and zero-shot protein fitness prediction, we solely evaluated MODIFY on the mutants with reliable fitness values.

High-order ParD3 mutants dataset. Ding et al.[7] experimentally assessed the mutation effects of antitoxin ParD3 in the ParD3-ParE3 complex. ParD3 forms an inert multimeric complex with the toxin ParE3 if co-expressed in *Escherichia coli*. Cells can grow if ParD3 and ParE3 interact, but the cell growth will be slowed down if the interaction is disrupted. The fitness of a given ParD3 variant reflects its interaction with the toxin ParE3, as measured by cell proliferation. This landscape covers $20^3 = 8,000$ mutants across three sites. The fitness values were normalized so that the wild-type fitness is 1.0 and the mean fitness of all variants with stop codons (i.e., truncated ParD3) is 0.0. During the evaluation of MODIFY for zero-shot protein fitness prediction, we only included variants without stop codons.

A.2 Co-optimization of the fitness and diversity of the library

Stochastic gradient ascent. At the library design stage of MODIFY, we co-optimize the expected fitness of sequences sampled by the library and the library's diversity:

$$\max_{p \in \mathcal{P}} \mathbb{E}_{\boldsymbol{x} \sim p(\boldsymbol{x})} \text{fitness}(\boldsymbol{x}) + \lambda \cdot \text{diversity}(p), \tag{1}$$

where $\mathcal{P}$ is the set of all possible libraries and $\lambda > 0$ is a coefficient that balances the fitness and diversity terms. The unconstrained optimization problem with respect to ϕ is:

$$\max_{\phi} J(\phi) = \max_{\phi} \mathbb{E}_{\boldsymbol{x} \sim p(\boldsymbol{x})}[f(\boldsymbol{x})] + \lambda \sum_{i=1}^{M} \alpha_i H(p_i), \tag{2}$$

where α_i is the parameter used for strengthening or reducing the diversity at residue i. We apply stochastic gradient ascent to solve this optimization problem. The gradient of $J(\phi)$ is given by

$$\nabla_{\phi_{i,j}} J(\phi) \approx \frac{1}{B} \sum_{b=1}^{B} f(\boldsymbol{x}^{(b)})(\delta_j(x_i^{(b)}) - p_{i,j}) - \lambda \alpha_i \sum_{j'=1}^{K} (1 + \log p_{i,j'}) p_{i,j'} (\delta_j(j') - p_{i,j}), \tag{3}$$

where B refers to the batch size and $x_i^{(b)}$ is the i-th AA of the b-th sequence in the batch.

We now show the derivation of this gradient. For the first term in Supplementary Eq. 2, we have

$$\begin{aligned}\nabla_{\phi} \mathbb{E}_{\boldsymbol{x} \sim p(\boldsymbol{x})}[f(\boldsymbol{x})] &= \nabla_{\phi} \sum_{\boldsymbol{x} \in \mathcal{X}} p(\boldsymbol{x}) f(\boldsymbol{x}) = \sum_{\boldsymbol{x} \in \mathcal{X}} f(\boldsymbol{x}) \nabla_{\phi} p(\boldsymbol{x}) \\ &= \sum_{\boldsymbol{x} \in \mathcal{X}} f(\boldsymbol{x}) p(\boldsymbol{x}) \nabla_{\phi} \log p(\boldsymbol{x}) = \mathbb{E}_{\boldsymbol{x} \sim p(\boldsymbol{x})}[f(\boldsymbol{x}) \nabla_{\phi} \log p(\boldsymbol{x})].\end{aligned} \tag{4}$$

Following Zhu et al.[8], we apply the Monte Carlo approximation to approximate the above gradient,

which takes the below form:

$$
\begin{aligned}
\nabla_{\phi_{i,j}} \mathbb{E}_{\boldsymbol{x}\sim p(\boldsymbol{x})}[f(\boldsymbol{x})] =& \mathbb{E}[f(\boldsymbol{x}) \nabla_{\phi_{i,j}} \log p(\boldsymbol{x})] \approx \frac{1}{B}\sum_{b=1}^{B} f(\boldsymbol{x}^{(b)}) \nabla_{\phi_{i,j}} \log p(\boldsymbol{x}^{(b)}) \\
=& \frac{1}{B}\sum_{b=1}^{B} f(\boldsymbol{x}^{(b)})(\delta_j(x_i^{(b)}) - p_{i,j}),
\end{aligned}
\tag{5}
$$

where B is the batch size, and $x_i^{(b)}$ is the i-th AA of the b-th sequence in a batch. For the second term in Supplementary Eq. 2, the gradient to the entropy of site i can be derived as

$$
\begin{aligned}
\nabla_{\phi_{i,j}} H(p_i) =& \nabla_{\phi_{i,j}} \sum_{j'=1}^{K} -p_{i,j'} \log p_{i,j'} = -\sum_{j'=1}^{K} \left(\nabla_{\phi_{i,j}} p_{i,j'} \log p_{i,j'} + p_{i,j'} \nabla_{\phi_{i,j}} \log p_{i,j'} \right) \\
=& -\sum_{j'=1}^{K} (p_{i,j'} \log p_{i,j'} \nabla_{\phi_{i,j}} \log p_{i,j'} + p_{i,j'} \nabla_{\phi_{i,j}} \log p_{i,j'}) \\
=& -\sum_{j'=1}^{K} (1 + \log p_{i,j'}) p_{i,j'} \nabla_{\phi_{i,j}} \log p_{i,j'} \\
=& -\sum_{j'=1}^{K} (1 + \log p_{i,j'}) p_{i,j'} (\delta_j(j') - p_{i,j}).
\end{aligned}
\tag{6}
$$

Exclusion of undesired AAs. The factorization of sequence probability as the product of site-wise AA probability, i.e., $p(\boldsymbol{x}) = \prod_{i=1}^{M} \sum_{k=1}^{K} \delta_k(x_i) p_{i,k}$, allows MODIFY to completely exclude some AAs at a site based on prior knowledge, such as experimentally confirmed loss-of-function mutations. Specifically, researchers can specify a set $\mathcal{U}_i$ of undesired AAs for position i (e.g., AAs that would destabilize structure), and MODIFY ensures that the final library will not include any AA from $\mathcal{U}_i$ at position i by adjusting the probability $p_{i,k}$ as

$$
p_{i,k} = \exp(\phi_{i,k} \odot S_{i,k}) / \sum_{k'} \exp(\phi_{i,k'} \odot S_{i,k}),
\tag{7}
$$

where $S \in \{0,1\}^{M\times K}$ is a binary mask matrix such that $S_{i,j} = 0$ if $j \in \mathcal{U}_i$ and one otherwise ($\forall i$), and $\odot$ represents element-wise multiplication. Since some site-wise distributions may have a support size smaller than $K = 20$ due to the masking, we re-scale the entropy in Eq. 3 to the same scale:

$$
\text{diversity}(p) = \sum_{i=1}^{M} [\log K / \log(K - |\mathcal{U}_i|)] H(p_i).
\tag{8}
$$

Parameter search space under MODIFY's default setting. Under the default setting, we varied the value of the parameter λ/M from a set of values and then selected the value of λ/M that produces the library with the maximum area (i.e., mean predicted fitness $\times$ diversity). For GB1 and cytochrome c, we varied the value of λ/M from 0 to 2, with increments of 0.01. For CreiLOV, we varied the value of λ/M from 0 to 1, with increments of 0.001.

A.3 Zero-shot protein fitness prediction

For zero-shot protein fitness prediction, MODIFY integrates four pre-trained unsupervised ML models to capture the evolutionary plausibility of protein sequences. Here, we describe our implementation of the four unsupervised models in detail.

Protein language model: In MODIFY, we integrated two PLMs, ESM-1v and ESM-2, for zero-shot protein fitness prediction. ESM-1v and ESM-2 have similar neural network architecture but were trained on different training sets (UniRef90 and UniRef50, respectively). ESM-1v is a collection of 5 pre-trained models on UniRef90 (esm1v_t33_650M_UR90S_{1, ..., 5}). For each variant, we first predict its fitness using the five ESM-1v models respectively and then average the predictions as the final predictions $s_{\mathrm{ESM-1v}}(\boldsymbol{x})$. For ESM-2, we use the pre-trained model esm2_t36_3B_UR50D for predicting the fitness $s_{\mathrm{ESM-2}}(\boldsymbol{x})$ for a given variant $\boldsymbol{x}$. The models and scripts of ESM-1v and ESM2 are downloaded from `https://github.com/facebookresearch/esm`.

Evolutionary coupling model: We integrated EVmutation[2] as the evolutionary coupling model in MODIFY. For a given parent protein, we first used the EVcouplings server (`https://evcouplings.org/`) to generate the multiple sequence alignment (MSA) and compute the evolutionary couplings model from the MSA. For MSA generation, we varied the bit score b from $\{0.1, 0.3, 0.5, 0.7\}$ while keeping other parameters as default. Notably, by default, columns in the MSA that have more than 30% of gaps (i.e., less than 70% of residues) will be excluded from the evolutionary couplings computation. Then, we selected the bit score b_{high}, which has the highest quality score as provided by the EVcouplings server, and the EVmutation model computed on the MSA generated by b_{high}. If the sites to be mutated in our library were excluded from the model's computation, we would increase the bit score (e.g., increase b from 0.3 to 0.5) to include the sites in the MSA. If no bit score from $\{0.1, 0.3, 0.5, 0.7\}$ satisfies this condition (e.g., CreiLOV), we would relax the position filter of no more than 30% gaps to include all sites and use the EVcouplings Python package[9] to recompute the evolutionary couplings with the MSA generated by bit score b_{high}. For benchmarking experiments on ProteinGym, we used the MSA pre-generated by ProteinGym and computed the evolutionary couplings model for each MSA using the EVcouplings Python package with default parameters.

Latent generative sequence model: For latent generative sequence models, we integrated EVE[10] into MODIFY. The probability of a sequence $\boldsymbol{x}$ is defined by marginalizing out the latent variable: $p(\boldsymbol{x}) = \int_z p(\boldsymbol{x}|z,\theta)p(z)dz$. This is approximated using the evidence lower bound (ELBO):

$$p(\boldsymbol{x}) \approx \mathbb{E}_q[\log p(\boldsymbol{x}|z,\theta)] - \mathrm{D}_{\mathrm{KL}}(q(z|\boldsymbol{x};\theta)||p(z)), \tag{9}$$

where both the conditional distribution $p(\boldsymbol{x}|z,\theta)$ and variational posterior $q(z|\boldsymbol{x};\theta)$ are modeled by neural networks. The protein fitness is characterized as the log-odds ratio: $s_{\mathrm{EVE}}(\boldsymbol{x}^{\mathrm{MT}}) = \log p(\boldsymbol{x}^{\mathrm{MT}}) - \log p(\boldsymbol{x}^{\mathrm{WT}})$. Following the GitHub repository of EVE (`https://github.com/OATML-Markslab/EVE`), we used the same MSA that was generated by the EVcouplings webserver for EVmutation. Following Frazer et al.[10], we set the sample size for computing the log-odds ratio as 2,000 and set $T = 0.2$ for correcting the biases in the MSA.

MSA-based PLM: As a hybrid PLM, MSA Transformer[11] combines global and local evolutionary information. Following Meier et al.[12] and Notin et al.[1], MSA Transformer scores the

fitness also as the log-odds ratio:

$$s_{\mathrm{MSATrans}}(\boldsymbol{x}^{\mathrm{MT}}) = \sum_{t \in T} \log p(x_t = x_t^{\mathrm{MT}} | \boldsymbol{x}_{\setminus T}; \mathrm{MSA}(\boldsymbol{x}^{\mathrm{WT}})) - \log p(x_t = x_t^{\mathrm{WT}} | \boldsymbol{x}_{\setminus T}; \mathrm{MSA}(\boldsymbol{x}^{\mathrm{WT}})), \tag{10}$$

where T is the set of mutated sites, $\setminus T$ represents the indices of other sites, and MSA$(\boldsymbol{x})$ represents the MSA of sequence $\boldsymbol{x}$. For MSA Transformer, we used the same MSA generated by the EVcouplings webserver. Following Rao et al.[11] and Notin et al.[1], we first filtered the MSA using HHFilter[13] and then sub-sampled the MSA to a size of 384 using the weight proposed by Hopf et al.[2] to reach optimal performances during inference. We sampled the MSA five times using five different random seeds and averaged the predictions from 5 different sub-sampled MSAs as the final fitness prediction $s_{\mathrm{MSATrans}}(\boldsymbol{x})$.

Ensemble fitness predictor: After we collected the predictions from the five unsupervised protein fitness predictors, we next ensemble them into the final predictions. As the fitness predictions from different models may have varying scales, we first performed a z-score transformation to normalize the predictions from different models to a comparable scale (zero mean and unit variance). Specifically, for each model, we first computed the mean μ and the standard deviation σ of its predictions for all the variants within the combinatorial search space, and we applied the transformation: $\tilde{s}(\boldsymbol{x}) = (s(\boldsymbol{x}) - \mu)/\sigma$. Then, we ensemble the predictions following Eq. 7, where specifically we have $\tilde{s}_{\mathrm{ESM}}(\boldsymbol{x}) = (\tilde{s}_{\mathrm{ESM-2}}(\boldsymbol{x}) + \tilde{s}_{\mathrm{ESM-1v}}(\boldsymbol{x}))/2$. Notably, after the z-score transformation, a random library with the uniform AA distribution at all sites would have a mean predicted fitness of 0.

A.4 Structure-based filter

As the four unsupervised protein fitness predictors integrated into MODIFY only leverage protein sequence and evolutionary information (MSAs) for fitness prediction, we further designed a structure-based filter as a quality check for MODIFY, aiming to improve the synthesizability of the variants in libraries designed by MODIFY (Fig. 1c). In specific, the structure-based filter in MODIFY is based on ESMFold pLDDT[14] for foldability and FoldX $\Delta\Delta G$[15] for structure stability. A variant would pass the filter if it meets any one of the two requirements (ESMFold $\mathrm{pLDDT} \geq c_1$ or FoldX $\Delta\Delta G \leq c_2$). The detailed implementation for each filter is described below.

Foldability filter. ESMFold predicts the 3D structures solely based on protein sequences and outputs per-atom pLDDT, reflecting the prediction confidence for the predicted structures. For each mutated sequence, MODIFY applies ESMFold to predict its structure and averages the pLDDT over the backbone carbon atoms. A higher pLDDT would indicate a higher prediction confidence of ESMFold for the given sequence and better foldability. In MODIFY, we used both the web server of ESMFold (`https://esmatlas.com/resources?action=fold`) and the local version of ESMFold (`https://github.com/facebookresearch/esm#esmfold`) for pLDDT calculations. We set the pLDDT threshold c_1 as the maximum of 85 and the median pLDDT of the variants in the searched landscape. Intuitively, a pLDDT higher than 85 would indicate a high foldability of the variant, and we would further increase the threshold if the majority of the variants' pLDDT is higher than 85. For GB1 and CreiLOV, we set c_1 as 85 and 88, respectively, as the medians of pLDDT were 82.0373 and 88.3853. For cytochrome *c*, as it would be computationally too expensive to screen the entire 6-site landscape, we randomly sampled 1,000 mutants from the landscape and set c_1 as 88 as the median was 87.7661.

Structure stability filter. FoldX $\Delta\Delta G$ (kcal/mol) measures the change in the change in Gibbs free energy between the wild-type (WT) structure and the mutant (MT) structure (i.e., $\Delta\Delta G_{\mathrm{MT}} = \Delta G_{\mathrm{MT}} - \Delta G_{\mathrm{WT}}$). The lower the $\Delta\Delta G$ is, the more stable the mutant structure is. For wild-type proteins with experimentally determined structures, we selected the commonly used PDB structures as the WT structures (PDB: 1PGA for GB1 and PDB: 3CP5 for cytochrome *c*). If the target proteins do not have experimentally determined structures or the experimentally determined structures miss certain sites to be mutated (PDB: 1N9L for CreiLOV), we used AlphaFold2[16] to predict the target protein's 3D structures. Before we used the predicted structures as the WT structure, we checked their quality by aligning the predicted structures with known PDB structures. For each mutant, we repeated the FoldX $\Delta\Delta G$ run five times to acquire robust results. The structure stability filter c_2 is set as the median of the mutants in the landscape. For GB1 and CreiLOV, we calculated $\Delta\Delta G$ for every mutant in the landscape and set c_2 as 25 kcal/mol and 3 kcal/mol, respectively, as the medians were 24.6212 kcal/mol and 2.9683 kcal/mol. For cytochrome *c*, as it would be computationally too expensive to perform $\Delta\Delta G$ calculations for every mutant in the landscape, we randomly sampled 1,000 mutants from the landscape for $\Delta\Delta G$ calculations and set c_2 as 4 kcal/mol as the median was 4.0227 kcal/mol.

A.5 High-quality starting library design for GB1

To evaluate the performance of MODIFY in designing high-quality libraries for protein engineering, we first applied MODIFY to design a starting library on the four-site combinatorial sequence space of GB1 and further performed an *in silico* ML-guided directed evolution experiment on the GB1 landscape. Here, we described our implementation in detail.

MODIFY's informed setting (MODIFY-informed). We applied the informed setting of MODIFY to design starting libraries for GB1 because we observed a notable difference between MODIFY's zero-shot predictions and the ground-truth fitness of single-mutation variants (Fig. 3f-g). This is an excellent example for the demonstration of how we can incorporate prior domain knowledge into MODIFY in addition to MODIFY's zero-shot protein fitness predictions, as the ground-truth single-mutation fitnesses of GB1 had been characterized in a work[17] prior to the experimental characterization of the combinatorial GB1 landscape. Under MODIFY's default setting, λ was set as 1.64 and we had $\alpha_i = 1/4, \forall i \in \{39, 40, 41, 54\}$. Under the informed setting, however, we aimed to increase the diversity at site 40, as guided by prior domain knowledge, and hence we fixed the values of $\lambda, \alpha_{39}, \alpha_{41}, \alpha_{54}$ and tuned only α_{40}. By increasing the value of α_{40}, the diversity of site 40 would increase, and the probability of D40 would drop. We here adopted a heuristic approach that uses the probability of the top-1 AAs at other sites as a reference and adjusts α_{40} accordingly. In specific, under the default setting, we observed that the leading AAs at other sites were L39, G41, and V54, which had probabilities of 29%, 53%, and 63%. We tuned α_{40} so that the probability of D40 is no larger than those probabilities. Eventually, we set $\lambda\alpha_{40}$ as 0.69 (i.e., $\alpha_{40} = 0.69/1.64$) so that D40 has a probability of 29%.

Library distribution evaluation. In our experiment, we evaluated the library distribution of MODIFY, MODIFY-informed, and NNK as shown in Fig. 3e. For each library distribution, we sampled 10^4 variants from the distribution (without removing the repeating variants) and evaluated the mean experimental fitness of the sampled variants.

Baseline methods implementation. For Exploitation, we first scored each variant within the search space using the zero-shot protein fitness predictor of MODIFY, and then we selected the 500

variants with the highest zero-shot protein fitness predictions to form the starting library. We re-calculated the MODIFY predictions (re-sampling the MSA for MSA Transformer) for 5 different seeds. For NNK, each site is characterized by the independent NNK distribution (N=A/C/G/T and K=G/T). We sampled 500 variants from the NNK distribution at the DNA level and then translated the DNA sequence to the protein sequence. As truncated GB1 variants (i.e., variants that have stop codons) have not been experimentally characterized, we excluded them during the evaluation of the library quality and the *in silico* MLDE experiment, which likely favored the NNK libraries as generally truncated proteins had low fitness. We repeated the sampling from NNK 5 times using different seeds. For FoldX, we performed the FoldX $\Delta\Delta G$ calculations for each variant, ranked the variants according to $\Delta\Delta G$ in the ascending order, and selected 500 variants with the lowest $\Delta\Delta G$ values. We repeated the FoldX $\Delta\Delta G$ run 5 times. For FuncLib, we used its web server (`https://ablift.weizmann.ac.il/step/fl_terms/`) for library construction on GB1 with default parameters. We used PDB 1PGA as the query structure and selected four amino acid positions (i.e., 39, 40, 41, and 54) to diversify. To maximize the size of the designed library for downstream MLDE, we did not perform clustering to the design library, resulting in a final library of 209 GB1 mutants.

Comparing MODIFY with DeCOIL and HotSpot Wizard. To ensure consistent comparison between MODIFY and DeCOIL, we used Triad $\Delta\Delta G$[18,19], a biophysical model for stability prediction, as the unsupervised fitness predictor for both approaches. We downloaded and used the Triad $\Delta\Delta G$ scores provided by Yang et al.[20] in the DeCOIL GitHub repository (`https://github.com/jsunn-y/DeCOIL`). Following Yang et al.[20], we implemented DeCOIL using three different values of p (0.1, 1, and 25) with the default random initialization of 240 templates and selected 10 unique templates with the top-weighted diffuse coverage (based on Hamming distance and $\sigma = 0.4$) for each value of p. For HotSpot Wizard v3.1, we designed libraries using its web tool (`https://loschmidt.chemi.muni.cz/hotspotwizard/`). We used PDB 1PGA as the query structure. To design combinatorial libraries for GB1, we manually chose V39, D40, G41, and V54 in the web tool for library construction. The Standard design mode was used based on the analysis of stability hot spots by structural flexibility, and amino acid frequency was used for the selection of amino acids. For each selected DeCOIL template and HotSpot Wizard template, we randomly sampled 500 variants and removed duplicated variants. We further removed variants with stop codons for DeCOIL and HotSpot Wizard, favoring DeCOIL and HotSpot Wizard during comparison. For MODIFY, we first normalized the Triad $\Delta\Delta G$ scores by z-score and then carried out the same co-optimization of the library fitness and diversity. In addition to the previously adopted values of λ/M (Supplementary Information A.2), we further varied the value of λ/M from 0 to 0.2 with increments of 0.001. Each MODIFY library corresponding to a λ value on the Pareto frontier generated 500 unique variants, with $\lambda = 0.396$ leading to the maximized area (zero-shot predicted fitness $\times$ diversity) under the Pareto frontier (Supplementary Fig. 4). We compared the libraries designed by DeCOIL, HotSpot Wizard, and MODIFY on the GB1 landscape, using mean experimental fitness and average entropy as the metrics (Supplementary Fig. 4).

t-SNE visualization. To visualize the combinatorial sequence search space of the GB1 protein in Figs. 4b–f, we encoded the variants within the landscape using ESM-2 (esm2_t36_3B_UR50D), which has a feature dimension of 2,560. We then used t-SNE to visualize the ESM-2 embeddings of the 160,000 variants from the search space in the 2D plane.

***In silico* MLDE experiment**. As one of the major goals for cold-start library design in protein

engineering is to collect training data as the guidance for downstream MLDE of the proteins, we have designed an *in silico* MLDE experiment on the GB1 landscape as a proof-of-concept and assess the ability of MODIFY's libraries for guiding the directed evolution. Using the experimentally characterized fitness data of the designed libraries, we first trained a supervised ML model to predict the variant's fitness from the sequence for each library and screened the remaining landscape with the trained ML model in search of high-fitness variants. We selected the simplest setting to demonstrate the intrinsic advantage of MODIFY's libraries. As there were four sites to be mutated on the GB1 landscape, we applied the one-hot encoding $w(\boldsymbol{x}) \in \{0,1\}^{4\times 20}$ for each variant $\boldsymbol{x}$, where $w(\boldsymbol{x})_{i,j}$ equals 1 if $\boldsymbol{x}$ has the j-th AA in the alphabet at the i-th site to be mutated otherwise 0. We then flattened $w(\boldsymbol{x})$ into a 1D vector with a length of 80. We trained the Random Forest Regressor model in the sklearn package as the supervised ML model to learn the sequence-to-function relationships under the default parameters. To have a fair comparison between the libraries, we constructed a withheld test set containing all of the variants that were not included in any of the designed libraries. Then, we screened the test set using the trained ML model and prioritized variants with top predicted fitness values for evaluation. Since all methods use the same ML model, a better prioritization performance suggests that the library used as training data is more informative for MLDE. We repeated the *in silico* MLDE experiment 25 times for each method (using 5 random seeds for library generation and 5 random seeds for ML model training for each designed library).

A.6 High-quality starting library design for CreiLOV

After we validated MODIFY on the landscape of GB1 for designing high-quality starting libraries, we further assessed MODIFY on the fitness landscape of CreiLOV[5] (Supplementary Note A.1) as an ablation study. Unlike the GB1 landscape that includes all possible variants for the four mutated positions (i.e., $20^4 = 160,000$ variants), the CreiLOV landscape is a combination of only 20 beneficial or neutral single mutations at 15 sites, which were identified in single-residue, site-saturation mutagenesis[5] (Supplementary Figs. 5a-b).

While the NNK approach is incapable of designing combinatorial libraries on this partial search space, MODIFY can be flexibly applied to design starting libraries on this landscape by excluding the undesired AAs at every site and only calculating diversity over the allowed AAs. Besides the default setting of MODIFY, we further included two libraries on the Pareto frontier: L_1, which has an average predicted zero-shot fitness of 95% of the maximum predicted zero-shot fitness, and L_2, which has an average entropy of 95% of the maximum average entropy (Supplementary Fig. 5c). We also compared MODIFY to the random method, which uniformly samples variants from the combinatorial search space of CreiLOV, the FoldX approach, and the Exploitation approach. For each approach, we designed a library of 500 non-repeating variants and repeated 5 times using different seeds.

We observed that the MODIFY's designed library strikes an optimal balance between the library's site-wise diversity and the mean predicted fitness even on the partial, 15-site landscape of CreiLOV (Supplementary Fig. 5c). By adjusting the parameter λ, MODIFY could slide through the Pareto frontier and provide the tradeoff between library fitness and diversity. We then used the ground truth fitness data of CreiLOV to evaluate MODIFY's designed libraries, where the fitness value of a CreiLOV variant represents its fluorescence. While the random approach achieved the highest diversity at the price of the lowest library fitness and Exploitation achieved the highest

library fitness at the price of the lowest library diversity, MODIFY's designed libraries achieved a controllable tradeoff between the high library fitness and the high library diversity (Supplementary Fig. 5d). For MODIFY (L_1), MODIFY, and MODIFY (L_2), the parameter λ were set as 0.3, 0.93, and 3, respectively, for all residue index i. As λ increased, the diversity of MODIFY's designed library increased while the library fitness decreased. Through this experiment, we further demonstrated the applicability of MODIFY as MODIFY is designed to be able to adapt to the landscapes of different proteins flexibly and to provide a controllable tradeoff for the users.

A.7 Experimental validation of MODIFY on engineering cytochrome *c*

Apart from the computational experiments, we applied MODIFY to designing a starting library for cytochrome c, and we evaluated the MODIFY's designed library against an NNK library in the wet lab for catalyzing new-to-biology reactions. Incorporating prior domain knowledge on engineering cytochrome c, we first designed a MODIFY library under the informed setting on 6 residues (75, 99, 100, 101, 102, and 103). Then, we expressed the cytochrome c variants designed by MODIFY and evaluated them for catalyzing the C–B bond formation reaction and C–Si bond formation reaction, using activity and enantioselectivity as the metrics. The computational design procedure and the experimental procedure are described below in detail.

A.7.1 Computational design procedure for MODIFY library

MODIFY's informed setting (MODIFY-informed). The residue-level diversity control of MODIFY enabled us to incorporate findings from prior efforts of directed evolution to inform our library design, in which we increased the diversity at residue 75 that harbors several beneficial amino acids for both reactions[21] and excluded specific amino acids (e.g., methionine at residue 100) that would inhibit the enzymatic activity in both insertion reactions[22]. While MODIFY's zero-shot predictions highly prioritized variants with the mutation V75M over other single mutations at site 75, prior directed evolution studies have identified V75T and V75R as important single mutations at site 75. Furthermore, as we observed that M75 has a high probability of 78% under the default setting of MODIFY (Fig. 5e; $\lambda = 1.44, \alpha_i = 1/6, \forall i \in \{75, 99, 100, 101, 102, 103\}$), we decided to increase the value of α_{75} so that the diversity at site 75 would be promoted. Similar to the approach we adopted for the experiment on GB1, we used the top-1 AAs at other sites as the reference. We tuned α_{75} so that the probability of M75 is as high as the second-highest top-1 AA, Q103, which has a probability of 59%. Eventually, we set $\lambda\alpha_{75}$ as 0.3 (i.e., α_{75}=0.3/1.44) so that M75 has a probability of 60% (Fig. 5f). The Pareto frontier of MODIFY's designs for cytochrome c is shown in Fig. 5d.

A.7.2 Experiment procedure for MODIFY library cloning and biocatalytic borylation and silylation reactions.

Oligo pool amplification. A DNA oligo pool (141 bp) containing 1,000 sequences designed by MODIFY was ordered from Twist Bioscience (South San Francisco, CA). The oligo pool was amplified according to the protocol provided by Twist Bioscience without modifications using the program detailed below.

Oligo pool amplification protocol. A stock solution of the oligo pool was resuspended in 10 mM Tris buffer, pH 8.0 to a final concentration of 20 ng/µL. The KAPA HiFi HotStart PCR

kit from Roche was used for amplification. In this process, 5 µL 5x KAPA HiFi buffer, 0.75 µL 10 mM dNTP, 0.75 µL 10 µM forward primer, 0.75 µL 10 µM reverse primer, 0.5 µL oligo pool, and 0.5 µL KAPA HiFi HotStart DNA polymerase (1 U/µL) were added into 25 µL reaction. The solution was mixed by gently tapping the PCR tube.

PCR cycling program: PCR reaction components are included in Supplementary Table 7, and PCR reaction conditions are included in Supplementary Table 8.

Forward primer: GTGGTCCAGTTTACATCATG

Reverse primer: GAATTGCACGTGCTTGTTCTT

Plasmid construction and transformation. pET–22b(+) was used as a cloning vector and Gibson assembly[23] was used to ligate DNA fragments. Following PCR amplification, the DNA fragments were cloned into a pET–22b(+) vector. Ligated plasmids were used to transform electrocompetent *E. cloni* BL21(DE3) cells (Lucigen) containing the cytochrome *c* maturation plasmid pEC86 (GenBank: OM367995.1). The pEC86 plasmid was provided by Prof. Kara Bren (University of Rochester).

MODIFY library sequencing. Following the transformation, the SOC culture was plated onto $LB_{amp/chlor}$ agar plates. Single colonies from $LB_{amp/chlor}$ agar plates were picked using sterile toothpicks and cultured in deep-well 96-well plates containing $LB_{amp/chlor}$ (400 µL) at 37 °C, 250 rpm shaking for 14 h. Glycerol stocks were prepared by mixing 80 µL starter culture with 50% v/v glycerol/water (80 µL) and stored in a –80 °C freezer. Frozen glycerol stocks were sent to Azenta Life Sciences (Burlington, MA) for sequencing.

Hemochrome assay for the determination of haem protein concentration[24,25]. In a conical tube, a solution of 0.2 M NaOH, 40% (v/v) pyridine, 0.5 mM $K_3Fe(CN)_6$ was prepared (Solution I: pyridine-NaOH-$K_3Fe(CN)_6$ solution). In another 1.5 mL centrifuge tube, a solution of 0.5 M sodium dithionite was prepared in 0.1 M NaOH. 500 µL of clarified lysate in M9-N minimal medium (abbreviated as M9-N buffer; pH 7.4) which contains 47.7 µM Na_2HPO_4, 22.0 µM KH_2PO_4, 8.6 µM NaCl, 2.0 µM $MgSO_4$, and 0.1 µM $CaCl_2$. and 500 µL of Solution I were transferred to a cuvette and carefully mixed. The UV-Vis spectrum of the oxidized Fe(III) state was recorded immediately. To the cuvette was then added 10 µL of the sodium dithionite solution (100 mg/mL). The cuvette was sealed with parafilm and the UV-Vis spectrum of the reduced Fe(II) state was recorded immediately. A cuvette containing 500 µL of M9-N and 500 µL Solution I was used as a reference for all absorbance measurements. Concentrations of cytochrome *c* were determined using a published extinction coefficient for heme *c*, ϵ_{550}(reduced) = 30.27 mM^{-1} cm^{-1}.

MODIFY and NNK library screening in 96-well plates for biocatalytic C–B bond formation. Single colonies were picked using sterile toothpicks from $LB_{amp/chlor}$ agar plates and grown in deep-well (2 mL) 96-well plates containing $LB_{amp/chlor}$ (400 µL) at 37 °C, 250 rpm shaking. After 16 h, aliquots of the overnight culture (60 µL) were transferred to deep-well 96-well plates containing $HB_{amp/chlor}$ (1 mL) using a 12-channel Eppendorf ResearchPlus multichannel pipette. Glycerol stocks of the libraries were prepared by mixing the starter culture (80 µL) with 50% v/v glycerol:water (80 µL). Glycerol stocks were stored at –78 °C in 96-well microplates. The expression cultures were shaken at 37 °C, 250 rpm for 3 h. The culture was placed on ice for 30 min, and isopropyl β-D-1-thiogalactopyranoside (IPTG) and 5-aminolevulinic acid (ALA) were added to final concentrations of 20 µM and 200 µM, respectively (total volume per well = 1.1 mL). The induced cultures were shaken at 20 °C, 220 rpm for 22 h. Cells were then pelleted (4,000 *g*, 5 min, 4 °C), resuspended in 370 µL M9-N buffer (pH = 7.4), and transferred to an anaerobic chamber. Inside the anaerobic chamber, to deep-well plates of cell suspensions were added a stock solution

of the NHC-BH_3 substrate (15 µL per well, 133 mM in MeCN) and the diazo compound (15 µL per well, 200 mM in MeCN). The final concentrations of the NHC-BH_3 and the diazo compound were 5 mM and 7.5 mM, respectively. The plates were then sealed with aluminum foil, shaken at 680 rpm on a Corning microplate shaker for 12 h, and then taken out of the anaerobic chamber. The reactions were quenched with hexanes:ethyl acetate (50:50 v/v, 600 µL) containing 1 mM mesitylene as the internal standard for HPLC analysis. The 96-well plates were sealed with silicone sealing mats and shaken vigorously to thoroughly mix the organic and aqueous layers. The plates were centrifuged (4,000 *g*, 5 min) to separate the aqueous and organic layers. 380 µL organic phase was transferred to 2.0 mL HPLC vials equipped with 500 µL inserts for HPLC analysis (Daicel IC column, 47% *i*-PrOH/Hexanes, 1.4 mL/min, t_R = 5.1 min (major), 6.6 min (minor)). HPLC traces of borane product are shown in Supplementary Figs. 7 and 8.

Analytical scale biocatalytic C–B bond forming reaction. 29 mL $HB_{amp/chlor}$ in a 125 mL flask was inoculated with an overnight culture (1 mL, $LB_{amp/chlor}$) of recombinant *E. cloni* BL21(DE3) cells containing a pET–22b(+) plasmid encoding the cytochrome *c* variant, and the pEC86 plasmid. The culture was shaken at 37 °C and 230 rpm until the OD_{600} was 0.7 (approximately 3 h). The culture was placed on ice for 30 min, and isopropyl β-D-1-thiogalactopyranoside (IPTG) and 5-aminolevulinic acid (ALA) were added to final concentrations of 20 µM and 200 µM, respectively, using a stock solution of 620 µM IPTG and 6.2 mM ALA in $HB_{amp/chlor}$ (1 mL of this stock solution was added to each expression culture). The incubator temperature was reduced to 20 °C, and the culture was shaken for 20 h at 150 rpm. Cells were collected by centrifugation (4,000 *g*, 5 min, 4 °C) and resuspended in M9-N buffer (pH = 7.4) to a target OD_{600} of 30. Following resuspension, 1 mL of the suspension was lysed using a QSonica Q500 ultrasonic homogenizer equipped with a stepped microtip (6 min total, 1 sec on, 1 sec off, 40% amplitude). The resulting lysed solution was centrifuged (21,000 *g*, 10 min, 4 °C) using an Eppendorf microcentrifuge 5425R to remove the cell debris. The supernatant (clarified lysate) was separated from the pellet and kept on ice for hemochrome assay to determine the haem protein concentration (the hemochrome assay protocol is described above).

In an anaerobic chamber, stock solutions of the NHC-BH_3 substrate (15 µL, 133 mM in MeCN), diazo compound (15 µL, 200 mM in MeCN), and sodium dithionite (40 µL, 0.1 M in degassed water) were added to a suspension of *E. coli* cells in M9-N buffer harbouring *Rma* cyt *c* variant (370 µL, adjusted to OD_{600} = 15) in a 2 mL vial. The vial was sealed and shaken at 680 rpm on a Corning microplate shaker at room temperature for 12 h. The vial was then taken out of the anaerobic chamber, and the reaction mixture was quenched with hexanes:ethyl acetate (1:1 v/v, 0.6 mL) containing 1 mM mesitylene as the internal standard. The reaction mixture was transferred to a microcentrifuge tube, vortexed (20 s), then centrifuged (21,000 *g*, 5 min) to completely separate the organic and aqueous layers. The organic layer (400 µL) was transferred to a 2.0 mL HPLC vial equipped with a 500 µL insert for HPLC analysis (Daicel IC column, 47% *i*-PrOH/Hexanes, 1.4 mL/min, 8 min).

Calibration curve development C–B bond formation. To a 1.5 mL microcentrifuge tube were added 400 µL of M9-N buffer solution. A stock solution of the authentic product in ethyl acetate and 600 µL extraction solvent hexanes:ethyl acetate (1:1 v/v) containing 1 mM mesitylene were added to the buffer. Final concentrations of the analyte were 0.0, 1.0, 2.0, 3.0, 4.0, 5.0, and 6.0 mM of, respectively. The mixture was vortexed (20 s for 3 times) and centrifuged (21000 *g*, 5 min) to separate the organic and aqueous layers. The organic layer was transferred to a vial with an insert for normal phase HPLC analysis (Daicel IC column, 47% *i*-PrOH/Hexanes, 1.4 mL/min,

8 min). The calibration curves detailed in Supplementary Fig. 9 product yield (y-axis) against the ratio of the peak area of product to the peak area of internal standard (x-axis). In the development of our calibration curves, care was taken such that our calibration curve samples were prepared in a way similar to enzymatic samples. The substrate calibration curve is made with the same method (Supplementary Fig. 10).

MODIFY and NNK library screening in 96-well plates for biocatalytic C–Si bond formation. Single colonies were picked using sterile toothpicks from $LB_{amp/chlor}$ agar plates and grown in deep-well (2 mL) 96-well plates containing $LB_{amp/chlor}$ (400 µL) at 37 °C, 250 rpm shaking. After 16 h, aliquots of the overnight culture (60 µL) were transferred to deep-well 96-well plates containing $HB_{amp/chlor}$ (1 mL) using a 12-channel Eppendorf ResearchPlus multichannel pipette. Glycerol stocks of the libraries were prepared by mixing the starter culture (80 µL) with 50% v/v glycerol:water (80 µL). Glycerol stocks were stored at –78 °C in 96-well microplates. The expression cultures were shaken at 37 °C, 250 rpm for 3 h. The culture was placed on ice for 30 min, and isopropyl β-D-1-thiogalactopyranoside (IPTG) and 5-aminolevulinic acid (ALA) were added to final concentrations of 20 µM and 200 µM, respectively (total volume per well = 1.1 mL). The induced cultures were shaken at 20 °C, 220 rpm for 22 h. Cells were then pelleted (4,000 *g*, 5 min, 4 °C), resuspended in 370 µL M9-N buffer (pH = 7.4), and transferred to an anaerobic chamber. Inside the anaerobic chamber, to deep-well plates of cell suspensions were added a stock solution of the $PhMe_2SiH$ substrate (15 µL per well, 133 mM in MeCN) and the diazo compound (15 µL per well, 200 mM in MeCN). The final concentrations of the $PhMe_2SiH$ and the diazo compound were 5 mM and 7.5 mM, respectively. The plates were then sealed with aluminum foil, shaken at 680 rpm on a Corning microplate shaker for 12 h, and then taken out of the anaerobic chamber. The reactions were quenched with hexanes:isopropanol (80:20 v/v, 600 µL) containing 1 mM mesitylene as the internal standard for HPLC analysis. The 96-well plates were sealed with silicone sealing mats and shaken vigorously to thoroughly mix the organic and aqueous layers. The plates were centrifuged (4,000 *g*, 5 min) to separate the aqueous and organic layers. 380 µL organic phase was transferred to 2.0 mL HPLC vials equipped with 500 µL inserts for HPLC analysis (CHIRALPAK IB N-5 column, 0.3% *i*-PrOH/Hexanes, 1.0 mL/min, 8 min, t_R = 5.7 (major), 6.4 (minor) min). HPLC traces of silane product are shown in Supplementary Figs. 11 and 12.

Analytical scale biocatalytic C–Si bond forming reaction. 29 mL $HB_{amp/chlor}$ in a 125 mL flask was inoculated with an overnight culture (1 mL, $LB_{amp/chlor}$) of recombinant *E. cloni* BL21(DE3) cells containing a pET–22b(+) plasmid encoding the cytochrome *c* variant, and the pEC86 plasmid. The culture was shaken at 37 °C and 230 rpm until the OD_{600} was 0.7 (approximately 3 h). The culture was placed on ice for 30 min, and isopropyl β-D-1-thiogalactopyranoside (IPTG) and 5-aminolevulinic acid (ALA) were added to final concentrations of 20 µM and 200 µM, respectively, using a stock solution of 620 µM IPTG and 6.2 mM ALA in $HB_{amp/chlor}$ (1 mL of this stock solution was added to each expression culture). The incubator temperature was reduced to 20 °C, and the culture was shaken for 20 h at 150 rpm. Cells were collected by centrifugation (4,000 *g*, 5 min, 4 °C) and resuspended in M9-N buffer (pH = 7.4) to a target OD_{600} of 15. Then the suspension was lysed using a QSonica Q500 ultrasonic homogenizer equipped with a stepped microtip (6 min total, 1 sec on, 1 sec off, 40% amplitude). The resulting lysed solution was centrifuged (21,000 *g*, 10 min, 4 °C) using an Eppendorf microcentrifuge 5425R to remove the cell debris. The supernatant (clarified lysate) was separated from the pellet and kept on ice for hemochrome assay to determine the haem protein concentration (the hemochrome assay protocol is described above).

In an anaerobic chamber, stock solutions of the $PhMe_2SiH$ substrate (10 µL, 800 mM in MeCN), diazo compound (10 µL, 400 mM in MeCN), and sodium dithionite (40 µL, 100 M in degassed water) were added to 370 µL lysate in a 2 mL vial. The vial was sealed and shaken at 680 rpm on a Corning microplate shaker at room temperature for 12 h. The vial was then taken out of the anaerobic chamber, and the reaction mixture was quenched with hexanes: *i*-Pr_2O (1:1 v/v, 0.6 mL) containing 1 mM mesitylene as the internal standard. The reaction mixture was transferred to a microcentrifuge tube, vortexed (20 s), and then centrifuged (21,000 g, 5 min) to completely separate the organic and aqueous layers. The organic layer (400 µL) was transferred to a 2.0 mL HPLC vial equipped with a 500 µL insert for HPLC analysis (CHIRALPAK IB N-5 column, 0.3% *i*-PrOH/Hexanes, 1.0 mL/min, 8 min).

Calibration curve development C–Si bond formation. To a 1.5 mL microcentrifuge tube were added 400 µL of M9-N buffer solution. A stock solution of the authentic product in ethyl acetate and 600 µL extraction solvent hexanes: *i*-Pr_2O (1:1 v/v) containing 1 mM mesitylene were added to the buffer. Final concentrations of the analyte were 0.0, 1.0, 2.0, 4.0, 8.0, and 12 mM of, respectively. The mixture was vortexed (20 s for 3 times) and centrifuged (21,000 g, 5 min) to separate the organic and aqueous layers. The organic layer was transferred to a vial with an insert for normal phase HPLC analysis (CHIRALPAK IB N-5 column, 0.3% i-PrOH/Hexanes, 1.0 mL/min, 8 min). The calibration curve in Supplementary Fig. 13 plots product yield (y-axis) against the ratio of the peak area of product to the peak area of internal standard (x-axis). In the development of our calibration curves, care was taken such that our calibration curve samples were prepared in a way similar to enzymatic samples.

Data processing. After we collected the activity and enatioselectivity data of the MODIFY and NNK libraries, we next processed our data to normalize the yield of all the variants between different plates. In each 96-well plate of NNK and MODIFY libraries, we included a total of 8 MMDTDT variants as a reference in wells A1, B2, C3, D4, E5, F6, G7 and H8. We first computed the average yield $\bar{y}$ of the reference variants on all plates. For each 96-well plate i, we computed the average yield $\bar{y}_i$ of this reference variant as the reference. Then, for each plate, we scaled the experimentally determined yields by $\bar{y}/\bar{y}_i$. While comparing the NNK library and the MODIFY library (Figs. 5i-j), data from these reference variants was not included.

A.8 Classical molecular dynamics (MD) simulations.

Classical MD simulations were performed to investigate the flexible loop dynamics of new enzyme mutants. The starting structure of the Fe carbene intermediates of the TDE variant was obtained from Protein Data Bank (PDB ID: 6CUN). Missing residues were added using the ModLoop server[26]. To generate cytochrome *c* variants, residues 75 and 99-103 were mutated using the Mutagenesis tool in PyMOL[27]. The geometries of substrates were optimized using the B3LYP functional[28,29] and 6-31G(d,p) basis set in Gaussian 16[30]. Substrates were then docked into cytochrome *c* variants using AutoDock[31] with the Lamarckian genetic algorithm. A grid box with dimensions of 40 Å, 40 Å, and 40 Å was used, whose center was set to be close to the carbene center. Docking parameters were set as follows: genetic algorithm run of 30, population size of 150, and 25 million energy evaluations. The best-scored pose from the docking calculation for each substrate was then used to construct the initial input geometry for classical MD simulations.

Classical MD simulations were carried out using the pmemd module[32] of the GPU-accelera-ted Amber 20 software[33]. The Amber ff14SB force field[34] was used in all classical MD simulations.

Parameters for substrates were generated using the general Amber force field (gaff2)[35]. Force field parameters for the Fe porphyrin carbene (IPC) species were generated using the MCPB.py module[36]. Using the Merz-Singh-Kollman scheme[37,38], RESP charge fitting[39] on electrostatic potential generated at the B3LYP/6-31G(d) level of theory was performed to generate partial charges at the open-shell singlet state, which was calculated to be the ground state of IPC intermediate[40]. Protonation states of enzyme residues were determined using the H++ server[41]. The enzyme was then put into a solvated cuboid box with periodic boundary condition using the TIP3P water model[42]. The minimum distance between the enzyme surface and the edge of the water box was set to 10 Å. Water molecules were treated with the SHAKE algorithm[43]. The system was neutralized by adding Na^+ counterions. Long-range electrostatic was calculated using the particle-mesh-Ewald method[44]. Lennard-Jones and electrostatic interaction cut-offs were set to 12 Å.

We first performed a 30,000-step energy minimization with positional restraints for the protein and the substrate by applying a force constant of 500 kcal·mol^{-1}·Å^{-2}. Next, the system was gradually heated from 0 K to 300 K in 200 ps, which was followed by an equilibration using the isothermal–isobaric ensemble (NPT) in the next 25 ns. Finally, production MD simulations were run in 1000 ns using the same conditions as the equilibration with a time step of 2 fs. In our MD simulations, to simulate the substrate near attack conformation[45] in the carbene insertion process and to prevent undesired substrate dissociation events, the carbene carbon and hydrogen atom distances were restrained in a range of 2.4–2.8 Å with a harmonic potential of 500 kcal·mol^{-1}·Å^{-2}. After the MD simulations, clustering analysis was carried out using the cpptraj module[46] to identify the most populated structure in 1000 ns of classical MD simulation. The RMSD value was used as the distance metric for clustering analysis.

To quantify the flexibility of each variant, B-factor values[47] (B_i, Å^2) were calculated for Cα atoms using root-mean-square fluctuation (ρ_i^{rmsf}) calculations implemented in cpptraj software:

$$B_i = \frac{8\pi^2}{3}(\rho_i^{rmsf})^2. \tag{11}$$

B Supplementary Figures

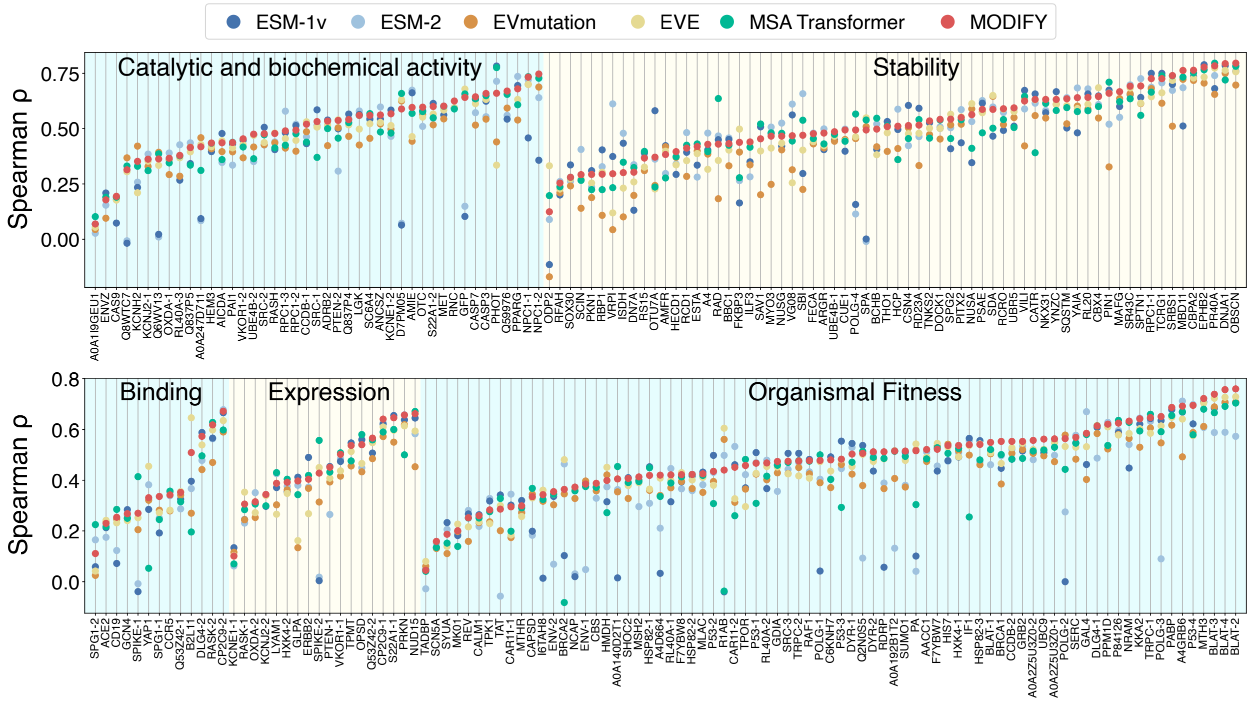

Supplementary Figure 1. MODIFY achieves accurate and robust zero-shot protein fitness prediction. The ensemble ML model of MODIFY was compared with five state-of-the-art unsupervised protein fitness predictors (ESM-1v, ESM-2, EVmutation, EVE, and MSA Transformer) for zero-shot protein fitness predictions. Comparison on the ProteinGym v1.0 benchmark, which contains 217 Deep Mutational Scanning (DMS) assays across diverse protein families, was reported using Spearman correlation as the evaluation metric.

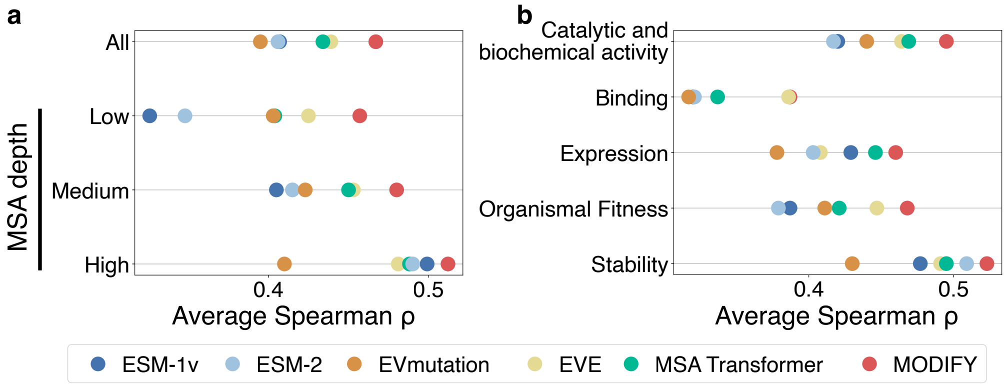

Supplementary Figure 2. MODIFY achieves accurate and robust zero-shot protein fitness prediction. The ensemble ML model of MODIFY was compared with five state-of-the-art unsupervised protein fitness predictors (ESM-1v, ESM-2, EVmutation, EVE, and MSA Transformer) for zero-shot protein fitness predictions on the ProteinGym v1.0 benchmark, which contains 217 Deep Mutational Scanning (DMS) assays across diverse protein families. **a,** The average performances of all methods on proteins with low, medium, and high MSA depths. **b,** The average performances of all methods on DMS assays with different function types (catalytic and biochemical activity, binding, expression, organismal fitness, and stability).

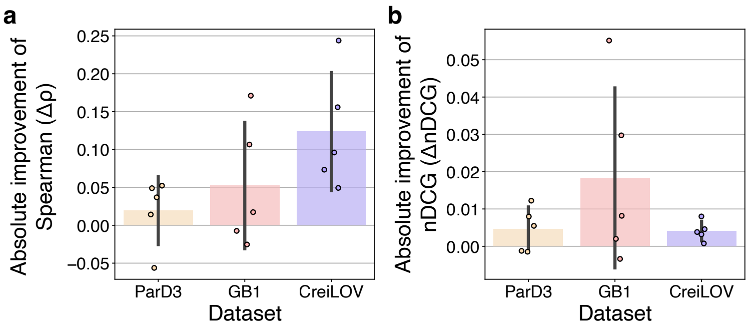

Supplementary Figure 3. MODIFY achieves accurate and robust zero-shot protein fitness prediction for high-order mutants. MODIFY was compared with five state-of-the-art unsupervised protein fitness predictors: ESM-1v, ESM-2, EVmutation, EVE, and MSA Transformer. **a–b,** Comparisons on predicting the fitness of the mutants from the landscapes of GB1, ParD3, and CreiLOV (covering 4, 3, and 15 residues, respectively), using the absolute improvement of Spearman correlation (**a**) and nDCG (**b**) of MODIFY over the mean performances of baseline methods as the evaluation metric. nDCG (Normalized Discounted Cumulative Gain) is a metric for assessing the ranking quality of a model: a high nDCG score would indicate that the model prioritizes variants with high fitness over variants with low fitness. The bar plots represented the mean ± SD of the data.

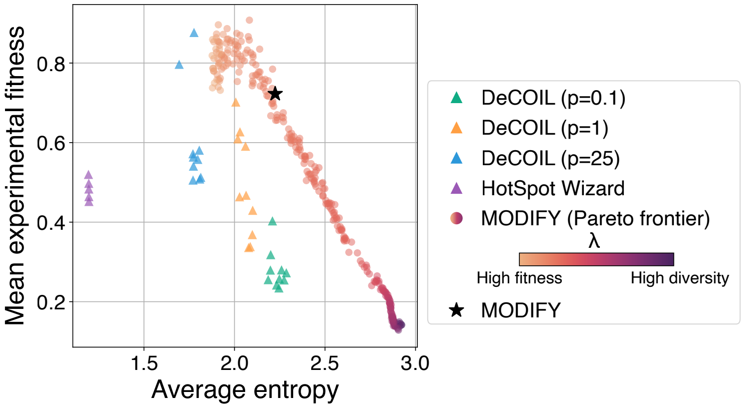

Supplementary Figure 4. MODIFY outperforms DeCOIL and HotSpot Wizard in combinatorial starting library design for GB1. MODIFY, DeCOIL, and HotSpot Wizard v3.1 were evaluated for designing a starting library for GB1 of size 500, using mean experimental fitness and average entropy as the metrics. For a fair comparison, Triad $\Delta\Delta G$ was used as the zero-shot prediction scores for both MODIFY and DeCOIL. Following Yang et al.[20], 10 unique DeCOIL templates with top-weighted diffuse coverages were selected from the 240 templates provided by each DeCOIL implementation as parameterized by p. For HotSpot Wizard, Standard design mode was employed, and five random seeds were used for sampling. As DeCOIL and HotSpot Wizard employed degenerate-codon libraries, duplicated variants were dropped for them. In contrast, MODIFY directly designed 500 unique variants.

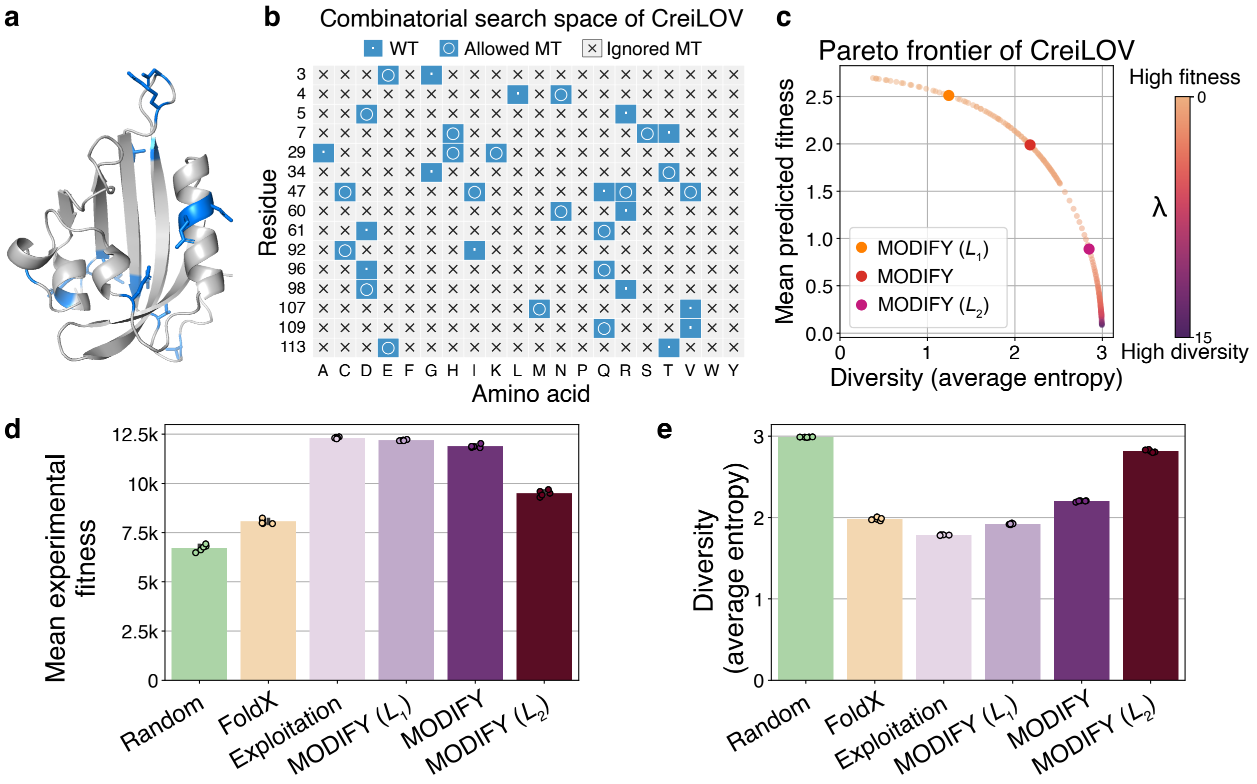

Supplementary Figure 5. MODIFY designs high-quality combinatorial starting libraries for CreiLOV. a, AlphaFold2 predicted 3D structure of CreiLOV. The residues mutated to create combinatorial libraries are colored in blue. **b,** The combinatorial search space of CreiLOV, unlike the GB1 landscape, only includes 20 single mutations that were previously determined beneficial or neutral (Supplementary Information A.1). **c,** The Pareto frontier of the CreiLOV library designs, with each point representing a library corresponding to a diversity strength λ. **d–e,** The mean experimental fitness and diversity (average entropy) of the designed libraries, each with 500 CreiLOV variants. In addition to MODIFY (default setting), MODIFY (L_1), which has an average predicted zero-shot fitness of 95% of the maximum predicted zero-shot fitness, and MODIFY (L_2), which has an average entropy of 95% of the maximum average entropy, were included. Random sampling, FoldX, and Exploitation were included as the baseline methods. The bar plots represented the mean $\pm$ SD over 5 independent repetitions.

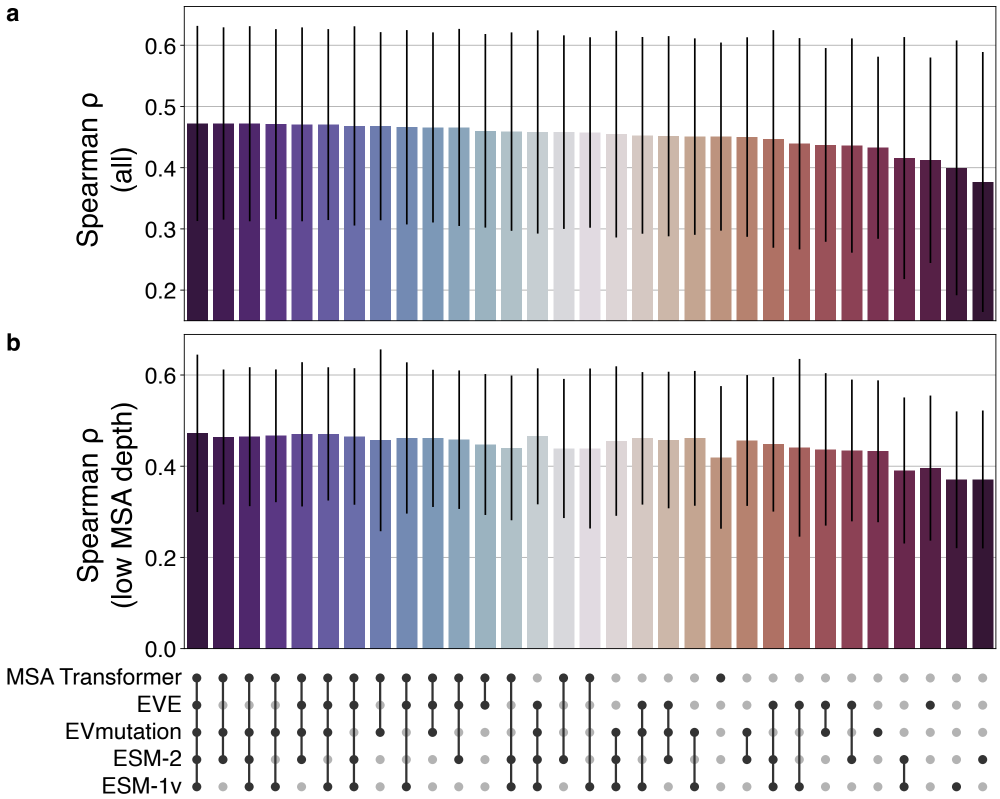

Supplementary Figure 6. MODIFY achieves accurate and robust zero-shot protein fitness prediction. MODIFY's ensemble-based zero-shot fitness prediction model was compared with different subset combinations of its constituent models (ESM-1v, ESM-2, EVmutation, EVE, and MSA Transformer). **a–b,** Comparison on the ProteinGym benchmark, which contains 87 Deep Mutational Scanning (DMS) assays across diverse protein families, using Spearman correlation averaged over all proteins (**a**) and over proteins with low MSA depths (**b**) as the evaluation metrics. For each combination, constituent models colored in black were included using the same β_i weight (Eq. 7). The bar plot represented the mean ± SD of the data.

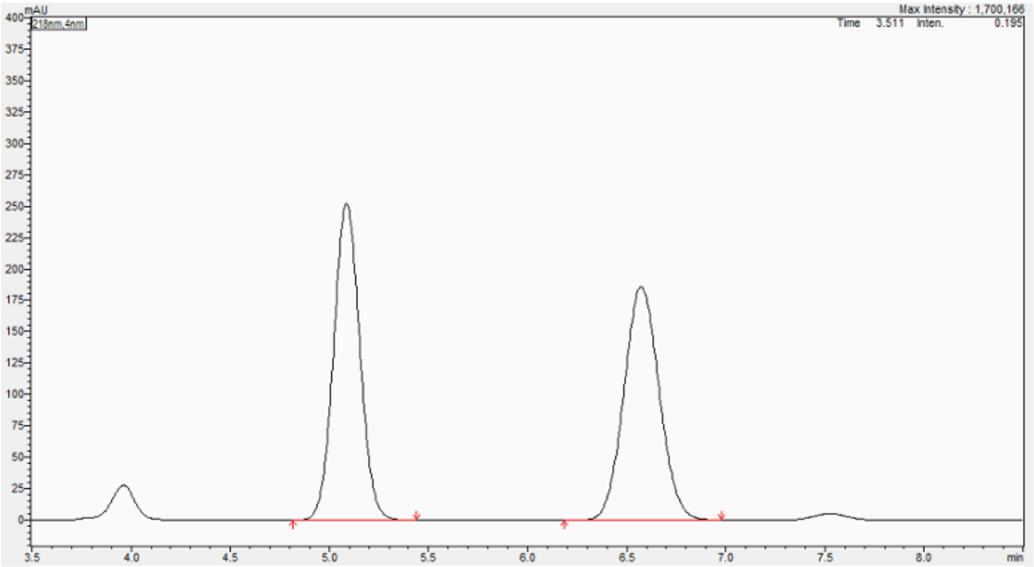

Peak#	Ret. Time	Peak Start	Peak End	Height	Area	Area%
1	5.087	4.816	5.440	252089	2307824	50.021
2	6.574	6.184	6.976	185785	2305883	49.979

Supplementary Figure 7. Borane product: racemic authentic sample (HPLC analysis).

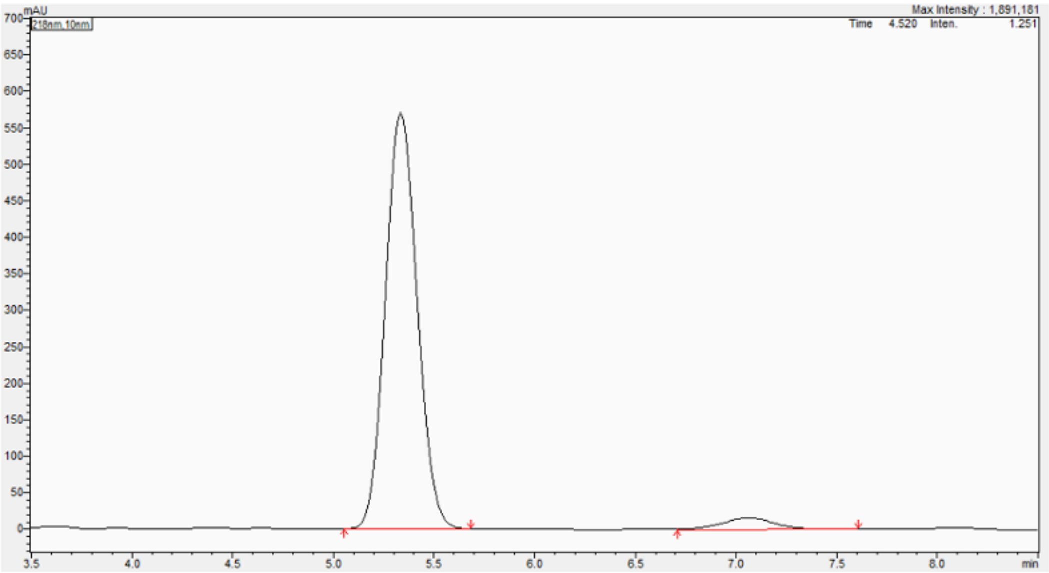

Peak#	Ret. Time	Peak Start	Peak End	Height	Area	Area%
1	5.328	5.056	5.685	570270	6327758	95.784
2	7.057	6.709	7.605	15683	278545	4.216

Supplementary Figure 8. Borane product: enantioenriched product obtained using MELQNQ variant: 96:4 e.r. (HPLC analysis).

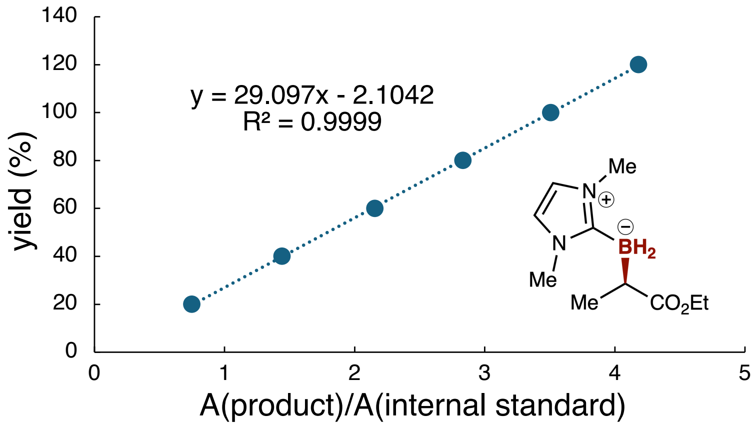

Supplementary Figure 9. The product calibration curve for C–B bond formation.

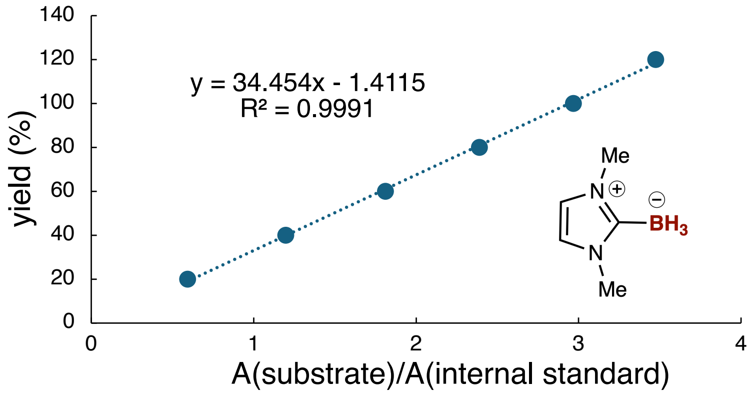

Supplementary Figure 10. The substrate calibration curve for C–B bond formation.

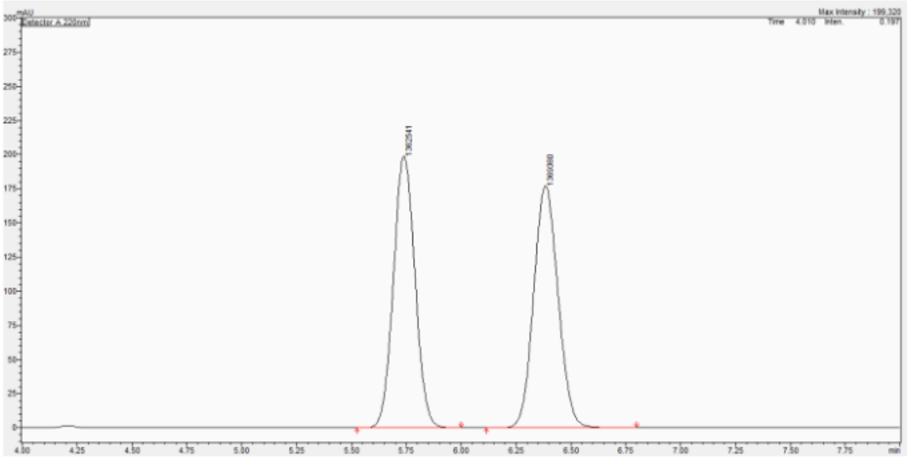

Peak#	Ret. Time	Peak Start	Peak End	Height	Area	Area%
1	5.740	5.525	6.000	199339	1362541	49.875
2	6.384	6.117	6.800	177496	1369380	50.125

Supplementary Figure 11. Silane product: racemic authentic sample (HPLC analysis).

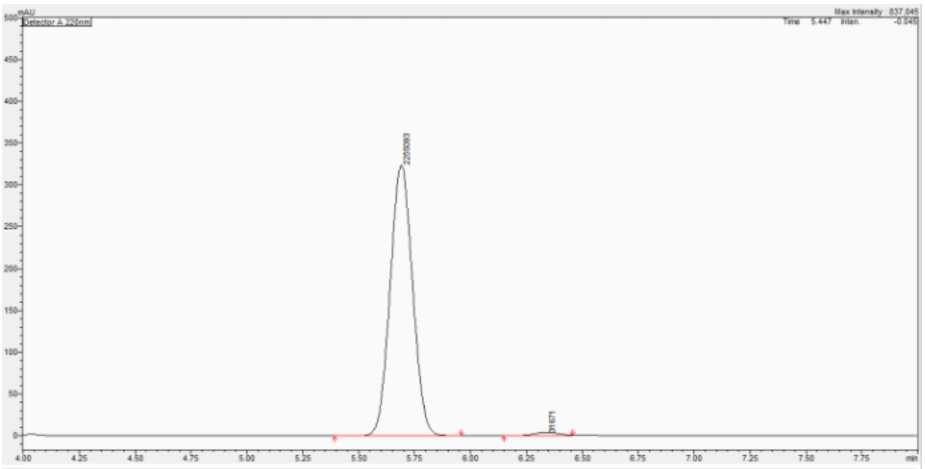

Peak#	Ret. Time	Peak Start	Peak End	Height	Area	Area%
1	5.691	5.392	5.958	324249	2255093	98.615
2	6.341	6.150	6.458	4032	31671	1.385

Supplementary Figure 12. Silane product: enantioenriched product obtained using TDE variant: 99:1 e.r. (HPLC analysis).

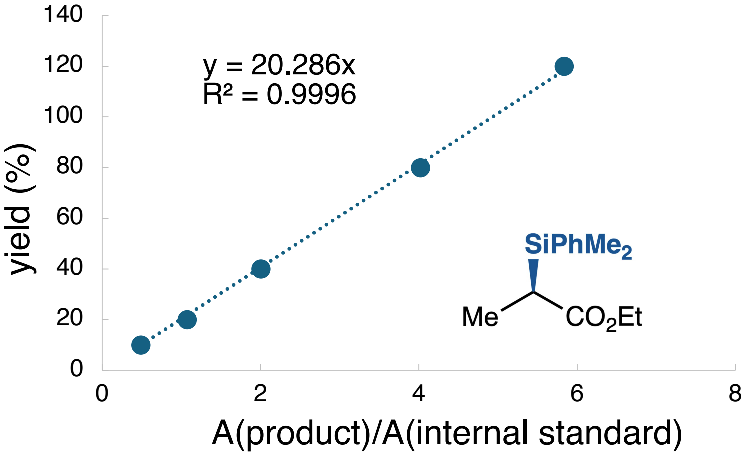

Supplementary Figure 13. The product calibration curve for C–Si bond formation.

C Supplementary Tables

Abbreviation	ProteinGym DMS dataset name	Abbreviation	ProteinGym DMS dataset name
A0A140D2T1	A0A140D2T1_ZIKV_Sourisseau_growth_2019	MTH3	MTH3_HAEAE_Rockah-Shmuel_2015
A0A192B1T2	A0A192B1T2_9HIV1_Haddox_2018	NCAP	NCAP_I34A1_Doud_2015
A0A1I9GEU1	A0A1I9GEU1_NEIME_Kennouche_2019	NRAM	NRAM_I33A0_Jiang_standard_2016
A0A2Z5U3Z0-1	A0A2Z5U3Z0_9INFA_Doud_2016	NUD15	NUD15_HUMAN_Suiter_2020
A0A2Z5U3Z0-2	A0A2Z5U3Z0_9INFA_Wu_2014	P53-1	P53_HUMAN_Giacomelli_NULL_Etoposide_2018
A4D664	A4D664_9INFA_Soh_CCL141_2019	P53-2	P53_HUMAN_Giacomelli_NULL_Nutlin_2018
A4GRB6	A4GRB6_PSEAI_Chen_2020	P53-3	P53_HUMAN_Giacomelli_WT_Nutlin_2018
A4	A4_HUMAN_Seuma_2021	P53-4	P53_HUMAN_Kotler_2018
AACC1	AACC1_PSEAI_Dandage_2018	P84126	P84126_THETH_Chan_2017
ADRB2	ADRB2_HUMAN_Jones_2020	PABP	PABP_YEAST_Melamed_2013
AMIE	AMIE_PSEAE_Wrenbeck_2017	PA	PA_I34A1_Wu_2015
B3VI55	B3VI55_LIPST_Klesmith_2015	POLG-1	POLG_CXB3N_Mattenberger_2021
BLAT-1	BLAT_ECOLX_Deng_2012	POLG-2	POLG_HCVJF_Qi_2014
BLAT-2	BLAT_ECOLX_Firnberg_2014	PTEN-1	PTEN_HUMAN_Matreyek_2021
BLAT-3	BLAT_ECOLX_Jacquier_2013	PTEN-2	PTEN_HUMAN_Mighell_2018
BLAT-4	BLAT_ECOLX_Stiffler_2015	Q2N0S5	Q2N0S5_9HIV1_Haddox_2018
BRCA1	BRCA1_HUMAN_Findlay_2018	Q59976	Q59976_STRSQ_Romero_2015
C6KNH7	C6KNH7_9INFA_Lee_2018	R1AB	R1AB_SARS2_Flynn_growth_2022
CALM1	CALM1_HUMAN_Weile_2017	RASH	RASH_HUMAN_Bandaru_2017
CAPSD	CAPSD_AAV2S_Sinai_substitutions_2021	REV	REV_HV1H2_Fernandes_2016
CCDB-1	CCDB_ECOLI_Adkar_2012	RL401-1	RL401_YEAST_Mavor_2016
CCDB-2	CCDB_ECOLI_Tripathi_2016	RL401-2	RL401_YEAST_Roscoe_2013
CP2C9-1	CP2C9_HUMAN_Amorosi_abundance_2021	RL401-3	RL401_YEAST_Roscoe_2014
CP2C9-2	CP2C9_HUMAN_Amorosi_activity_2021	SC6A4	SC6A4_HUMAN_Young_2021
DLG4-1	DLG4_HUMAN_Faure_2021	SCN5A	SCN5A_HUMAN_Glazer_2019
DLG4-2	DLG4_RAT_McLaughlin_2012	SPG1	SPG1_STRSG_Olson_2014
DYR	DYR_ECOLI_Thompson_plusLon_2019	SPIKE-1	SPIKE_SARS2_Starr_bind_2020
ENV-1	ENV_HV1B9_DuenasDecamp_2016	SPIKE-2	SPIKE_SARS2_Starr_expr_2020
ENV-2	ENV_HV1BR_Haddox_2016	SRC	SRC_HUMAN_Ahler_CD_2019
ESTA	ESTA_BACSU_Nutschel_2020	SUMO1	SUMO1_HUMAN_Weile_2017
F7YBW8	F7YBW8_MESOW_Aakre_2015	SYUA	SYUA_HUMAN_Newberry_2020
GAL4	GAL4_YEAST_Kitzman_2015	TADBP	TADBP_HUMAN_Bolognesi_2019
GCN4	GCN4_YEAST_Staller_induction_2018	TAT	TAT_HV1BR_Fernandes_2016
GFP	GFP_AEQVI_Sarkisyan_2016	TPK1	TPK1_HUMAN_Weile_2017
GRB2	GRB2_HUMAN_Faure_2021	TPMT	TPMT_HUMAN_Matreyek_2018
HIS7	HIS7_YEAST_Pokusaeva_2019	TPOR	TPOR_HUMAN_Bridgford_S505N_2020
HSP82-1	HSP82_YEAST_Flynn_2019	TRPC-1	TRPC_SACS2_Chan_2017
HSP82-2	HSP82_YEAST_Mishra_2016	TRPC-2	TRPC_THEMA_Chan_2017
I6TAH8	I6TAH8_I68A0_Doud_2015	UBC9	UBC9_HUMAN_Weile_2017
IF1	IF1_ECOLI_Kelsic_2016	UBE4B	UBE4B_MOUSE_Starita_2013
KCNH2	KCNH2_HUMAN_Kozek_2020	VKOR1-1	VKOR1_HUMAN_Chiasson_abundance_2020
KKA2	KKA2_KLEPN_Melnikov_2014	VKOR1-2	VKOR1_HUMAN_Chiasson_activity_2020
MK01	MK01_HUMAN_Brenan_2016	YAP1	YAP1_HUMAN_Araya_2012
MSH2	MSH2_HUMAN_Jia_2020		

Supplementary Table 1. The abbreviations for DMS dataset names in the ProteinGym substitution benchmark dataset. For formatting purposes, the DMS dataset names used in the ProteinGym dataset were abbreviated in Fig. 2. Digit suffixes were used to further distinguish between different DMS studies targeting the same protein.

Abbreviation	ProteinGym DMS dataset name	Abbreviation	ProteinGym DMS dataset name	Abbreviation	ProteinGym DMS dataset name
A0A140D2T1	A0A140D2T1_ZIKV_Sourisseau_2019	HIS7	HIS7_YEAST_Pokusaeva_2019	Q837P5	Q837P5_ENTFA_Meier_2023
A0A192B1T2	A0A192B1T2_9HIV1_Haddox_2018	HMDH	HMDH_HUMAN_Jiang_2019	Q8WTC7	Q8WTC7_9CNID_Somermeyer_2022
A0A1I9GEU1	A0A1I9GEU1_NEIME_Kennouche_2019	HSP82-1	HSP82_YEAST_Cote-Hammarlof_2020_growth-H2O2	R1AB	R1AB_SARS2_Flynn_2022
A0A247D711	A0A247D711_LISMN_Stadelmann_2021	HSP82-2	HSP82_YEAST_Flynn_2019	RAD	RAD_ANTMA_Tsuboyama_2023_2CJJ
A0A2Z5U3Z0-1	A0A2Z5U3Z0_9INFA_Doud_2016	HSP82-3	HSP82_YEAST_Mishra_2016	RAF1	RAF1_HUMAN_Zinkus-Boltz_2019
A0A2Z5U3Z0-2	A0A2Z5U3Z0_9INFA_Wu_2014	HXK4-1	HXK4_HUMAN_Gersing_2022_activity	RASH	RASH_HUMAN_Bandaru_2017
A4D664	A4D664_9INFA_Soh_2019	HXK4-2	HXK4_HUMAN_Gersing_2023_abundance	RASK-1	RASK_HUMAN_Weng_2022_abundance
A4GRB6	A4GRB6_PSEAI_Chen_2020	I6TAH8	I6TAH8_I68A0_Doud_2015	RASK-2	RASK_HUMAN_Weng_2022_binding-DARPin_K55
A4	A4_HUMAN_Seuma_2022	IF1	IF1_ECOLI_Kelsic_2016	RBP1	RBP1_HUMAN_Tsuboyama_2023_2KWH
AACC1	AACC1_PSEAI_Dandage_2018	ILF3	ILF3_HUMAN_Tsuboyama_2023_2L33	RCD1	RCD1_ARATH_Tsuboyama_2023_5OAO
ACE2	ACE2_HUMAN_Chan_2020	ISDH	ISDH_STAAW_Tsuboyama_2023_2LHR	RCRO	RCRO_LAMBD_Tsuboyama_2023_1ORC
ADRB2	ADRB2_HUMAN_Jones_2020	KCNE1-1	KCNE1_HUMAN_Muhammad_2023_expression	RD23A	RD23A_HUMAN_Tsuboyama_2023_1IFY
AICDA	AICDA_HUMAN_Gajula_2014_3cycles	KCNE1-2	KCNE1_HUMAN_Muhammad_2023_function	RDRP	RDRP_I33A0_Li_2023
AMFR	AMFR_HUMAN_Tsuboyama_2023_4G3O	KCNH2	KCNH2_HUMAN_Kozek_2020	REV	REV_HV1H2_Fernandes_2016
AMIE	AMIE_PSEAE_Wrenbeck_2017	KCNJ2-1	KCNJ2_MOUSE_Coyote-Maestas_2022_function	RFAH	RFAH_ECOLI_Tsuboyama_2023_2LCL
ANCSZ	ANCSZ_Hobbs_2022	KCNJ2-2	KCNJ2_MOUSE_Coyote-Maestas_2022_surface	RL20	RL20_AQUAE_Tsuboyama_2023_1GYZ
ARGR	ARGR_ECOLI_Tsuboyama_2023_1AOY	KKA2	KKA2_KLEPN_Melnikov_2014	RL40A-1	RL40A_YEAST_Mavor_2016
B2L11	B2L11_HUMAN_Dutta_2010_binding-Mcl-1	LGK	LGK_LIPST_Klesmith_2015	RL40A-2	RL40A_YEAST_Roscoe_2013
BBC1	BBC1_YEAST_Tsuboyama_2023_1TG0	LYAM1	LYAM1_HUMAN_Elazar_2016	RL40A-3	RL40A_YEAST_Roscoe_2014
BCHB	BCHB_CHLTE_Tsuboyama_2023_2KRU	MAFG	MAFG_MOUSE_Tsuboyama_2023_1K1V	RNC	RNC_ECOLI_Weeks_2023
BLAT-1	BLAT_ECOLX_Deng_2012	MBD11	MBD11_ARATH_Tsuboyama_2023_6ACV	RPC1-1	RPC1_BP434_Tsuboyama_2023_1R69
BLAT-2	BLAT_ECOLX_Firnberg_2014	MET	MET_HUMAN_Estevam_2023	RPC1-2	RPC1_LAMBD_Li_2019_high-expression
BLAT-3	BLAT_ECOLX_Jacquier_2013	MK01	MK01_HUMAN_Brenan_2016	RPC1-3	RPC1_LAMBD_Li_2019_low-expression
BLAT-4	BLAT_ECOLX_Stiffler_2015	MLAC	MLAC_ECOLI_MacRae_2023	RS15	RS15_GEOSE_Tsuboyama_2023_1A32
BRCA1	BRCA1_HUMAN_Findlay_2018	MSH2	MSH2_HUMAN_Jia_2020	S22A1-1	S22A1_HUMAN_Yee_2023_abundance
BRCA2	BRCA2_HUMAN_Erwood_2022_HEK293T	MTH3	MTH3_HAEAE_RockahShmuel_2015	S22A1-2	S22A1_HUMAN_Yee_2023_activity
C6KNH7	C6KNH7_9INFA_Lee_2018	MTHR	MTHR_HUMAN_Weile_2021	SAV1	SAV1_MOUSE_Tsuboyama_2023_2YSB
CALM1	CALM1_HUMAN_Weile_2017	MYO3	MYO3_YEAST_Tsuboyama_2023_2BTT	SBI	SBI_STAAM_Tsuboyama_2023_2JVG
CAPSD	CAPSD_AAV2S_Sinai_2021	NCAP	NCAP_I34A1_Doud_2015	SC6A4	SC6A4_HUMAN_Young_2021
CAR11-1	CAR11_HUMAN_Meitlis_2020_gof	NKX31	NKX31_HUMAN_Tsuboyama_2023_2L9R	SCIN	SCIN_STAAR_Tsuboyama_2023_2QFF
CAR11-2	CAR11_HUMAN_Meitlis_2020_lof	NPC1-1	NPC1_HUMAN_Erwood_2022_HEK293T	SCN5A	SCN5A_HUMAN_Glazer_2019
CAS9	CAS9_STRP1_Spencer_2017_positive	NPC1-2	NPC1_HUMAN_Erwood_2022_RPE1	SDA	SDA_BACSU_Tsuboyama_2023_1PV0
CASP3	CASP3_HUMAN_Roychowdhury_2020	NRAM	NRAM_I33A0_Jiang_2016	SERC	SERC_HUMAN_Xie_2023
CASP7	CASP7_HUMAN_Roychowdhury_2020	NUD15	NUD15_HUMAN_Suiter_2020	SHOC2	SHOC2_HUMAN_Kwon_2022
CATR	CATR_CHLRE_Tsuboyama_2023_2AMI	NUSA	NUSA_ECOLI_Tsuboyama_2023_1WCL	SOX30	SOX30_HUMAN_Tsuboyama_2023_7JJK
CBPA2	CBPA2_HUMAN_Tsuboyama_2023_1O6X	NUSG	NUSG_MYCTU_Tsuboyama_2023_2MI6	SPA	SPA_STAAU_Tsuboyama_2023_1LP1
CBS	CBS_HUMAN_Sun_2020	OBSCN	OBSCN_HUMAN_Tsuboyama_2023_1V1C	SPG1-1	SPG1_STRSG_Olson_2014
CBX4	CBX4_HUMAN_Tsuboyama_2023_2K28	ODP2	ODP2_GEOSE_Tsuboyama_2023_1W4G	SPG1-2	SPG1_STRSG_Wu_2016
CCDB-1	CCDB_ECOLI_Adkar_2012	OPSD	OPSD_HUMAN_Wan_2019	SPG2	SPG2_STRSG_Tsuboyama_2023_5UBS
CCDB-2	CCDB_ECOLI_Tripathi_2016	OTC	OTC_HUMAN_Lo_2023	SPIKE-1	SPIKE_SARS2_Starr_2020_binding
CCR5	CCR5_HUMAN_Gill_2023	OTU7A	OTU7A_HUMAN_Tsuboyama_2023_2L2D	SPIKE-2	SPIKE_SARS2_Starr_2020_expression
CD19	CD19_HUMAN_Klesmith_2019_FMC_singles	OXDA-1	OXDA_RHOTO_Vanella_2023_activity	SPTN1	SPTN1_CHICK_Tsuboyama_2023_1TUD
CP2C9-1	CP2C9_HUMAN_Amorosi_2021_abundance	OXDA-2	OXDA_RHOTO_Vanella_2023_expression	SQSTM	SQSTM_MOUSE_Tsuboyama_2023_2RRU
CP2C9-2	CP2C9_HUMAN_Amorosi_2021_activity	P53-1	P53_HUMAN_Giacomelli_2018_Null_Etoposide	SR43C	SR43C_ARATH_Tsuboyama_2023_2N88
CSN4	CSN4_MOUSE_Tsuboyama_2023_1UFM	P53-2	P53_HUMAN_Giacomelli_2018_Null_Nutlin	SRBS1	SRBS1_HUMAN_Tsuboyama_2023_2O2W
CUE1	CUE1_YEAST_Tsuboyama_2023_2MYX	P53-3	P53_HUMAN_Giacomelli_2018_WT_Nutlin	SRC-1	SRC_HUMAN_Ahler_2019
D7PM05	D7PM05_CLYGR_Somermeyer_2022	P53-4	P53_HUMAN_Kotler_2018	SRC-2	SRC_HUMAN_Chakraborty_2023_binding-DAS_25uM
DLG4-1	DLG4_HUMAN_Faure_2021	P84126	P84126_THETH_Chan_2017	SRC-3	SRC_HUMAN_Nguyen_2022
DLG4-2	DLG4_RAT_McLaughlin_2012	PABP	PABP_YEAST_Melamed_2013	SUMO1	SUMO1_HUMAN_Weile_2017
DN7A	DN7A_SACS2_Tsuboyama_2023_1JIC	PAI1	PAI1_HUMAN_Huttinger_2021	SYUA	SYUA_HUMAN_Newberry_2020
DNJA1	DNJA1_HUMAN_Tsuboyama_2023_2LO1	PA	PA_I34A1_Wu_2015	TADBP	TADBP_HUMAN_Bolognesi_2019
DOCK1	DOCK1_MOUSE_Tsuboyama_2023_2M0Y	PHOT	PHOT_CHLRE_Chen_2023	TAT	TAT_HV1BR_Fernandes_2016
DYR-1	DYR_ECOLI_Nguyen_2023	PIN1	PIN1_HUMAN_Tsuboyama_2023_1I6C	TCRG1	TCRG1_MOUSE_Tsuboyama_2023_1E0L
DYR-2	DYR_ECOLI_Thompson_2019	PITX2	PITX2_HUMAN_Tsuboyama_2023_2L7M	THO1	THO1_YEAST_Tsuboyama_2023_2WQG
ENVZ	ENVZ_ECOLI_Ghose_2023	PKN1	PKN1_HUMAN_Tsuboyama_2023_1URF	TNKS2	TNKS2_HUMAN_Tsuboyama_2023_5JRT
ENV-1	ENV_HV1B9_DuenasDecamp_2016	POLG-1	POLG_CXB3N_Mattenberger_2021	TPK1	TPK1_HUMAN_Weile_2017
ENV-2	ENV_HV1BR_Haddox_2016	POLG-2	POLG_DEN26_Suphatrakul_2023	TPMT	TPMT_HUMAN_Matreyek_2018
EPHB2	EPHB2_HUMAN_Tsuboyama_2023_1F0M	POLG-3	POLG_HCVJF_Qi_2014	TPOR	TPOR_HUMAN_Bridgford_2020
ERBB2	ERBB2_HUMAN_Elazar_2016	POLG-4	POLG_PESV_Tsuboyama_2023_2MXD	TRPC-1	TRPC_SACS2_Chan_2017
ESTA	ESTA_BACSU_Nutschel_2020	PPARG	PPARG_HUMAN_Majithia_2016	TRPC-2	TRPC_THEMA_Chan_2017
F7YBW7	F7YBW7_MESOW_Ding_2023	PPM1D	PPM1D_HUMAN_Miller_2022	UBC9	UBC9_HUMAN_Weile_2017
F7YBW8	F7YBW8_MESOW_Aakre_2015	PR40A	PR40A_HUMAN_Tsuboyama_2023_1UZC	UBE4B-1	UBE4B_HUMAN_Tsuboyama_2023_3L1X
FECA	FECA_ECOLI_Tsuboyama_2023_2D1U	PRKN	PRKN_HUMAN_Clausen_2023	UBE4B-2	UBE4B_MOUSE_Starita_2013
FKBP3	FKBP3_HUMAN_Tsuboyama_2023_2KFV	PSAE	PSAE_SYNP2_Tsuboyama_2023_1PSE	UBR5	UBR5_HUMAN_Tsuboyama_2023_1I2T
GAL4	GAL4_YEAST_Kitzman_2015	PTEN-1	PTEN_HUMAN_Matreyek_2021	VG08	VG08_BPP22_Tsuboyama_2023_2GP8
GCN4	GCN4_YEAST_Staller_2018	PTEN-2	PTEN_HUMAN_Mighell_2018	VILI	VILI_CHICK_Tsuboyama_2023_1YU5
GDIA	GDIA_HUMAN_Silverstein_2021	Q2N0S5	Q2N0S5_9HIV1_Haddox_2018	VKOR1-1	VKOR1_HUMAN_Chiasson_2020_abundance
GFP	GFP_AEQVI_Sarkisyan_2016	Q53Z42-1	Q53Z42_HUMAN_McShan_2019_binding-TAPBPR	VKOR1-2	VKOR1_HUMAN_Chiasson_2020_activity
GLPA	GLPA_HUMAN_Elazar_2016	Q53Z42-2	Q53Z42_HUMAN_McShan_2019_expression	VRPI	VRPI_BPT7_Tsuboyama_2023_2WNM
GRB2	GRB2_HUMAN_Faure_2021	Q59976	Q59976_STRSQ_Romero_2015	YAIA	YAIA_ECOLI_Tsuboyama_2023_2KVT
HCP	HCP_LAMBD_Tsuboyama_2023_2L6Q	Q6WV13	Q6WV13_9MAXI_Somermeyer_2022	YAP1	YAP1_HUMAN_Araya_2012
HECD1	HECD1_HUMAN_Tsuboyama_2023_3DKM	Q837P4	Q837P4_ENTFA_Meier_2023	YNZC	YNZC_BACSU_Tsuboyama_2023_2JVD
HEM3	HEM3_HUMAN_Loggerenberg_2023				

Supplementary Table 2. The abbreviations for DMS dataset names in the ProteinGym v1.0 substitution benchmark dataset. For formatting purposes, the DMS dataset names used in the ProteinGym v1.0 dataset were abbreviated in Supplementary Fig. 1. Digit suffixes were used to further distinguish between different DMS studies targeting the same protein.

entry	plate	variant (75,99-103)	yield (%)	e.r.	entry	plate	variant (75,99-103)	yield (%)	e.r.
1	1	MMLTDQ	89	95:5	81	3	MTVPNQ	81	96:4
2	1	MLYPPT	88	96:4	82	3	MPQPNQ	78	95:5
3	1	MVYGDQ	89	95:5	83	3	MQVPTQ	74	97:3
4	1	MGAANQ	88	95:5	84	3	APIANQ	81	88:12
5	1	MELQNQ	86	95:5	85	3	SNAPPT	81	83:17
6	1	MPEPNQ	86	95:5	86	3	MRFPDQ	70	95:5
7	1	MLLTAQ	87	94:6	87	3	ALLGQT	80	84:16
8	1	MVKPNP	85	96:4	88	3	SRFTDM	75	84:16
9	1	MRNPNQ	83	96:4	89	3	MRWPWQ	65	95:5
10	1	MPIPDQ	82	95:5	90	3	MLLSDA	63	95:5
11	1	MPIPDQ	81	95:5	91	3	LQIPNQ	76	78:22
12	1	MDEPPQ	81	95:5	92	3	MPAEFQ	62	95:5
13	1	MPIPGQ	81	95:5	93	3	MAIPAQ	62	96:4
14	1	MVAAPL	80	93:7	94	3	MPFPVQ	63	94:6
15	1	SFLTNQ	85	86:14	95	3	MPEPNQ	61	96:4
16	1	MALMNM	76	94:6	96	3	MHLRNN	61	94:6
17	1	MQLVDQ	74	96:4	97	3	KPWPNY	70	82:18
18	1	MPNTNV	72	94:6	98	3	MIITNQ	60	95:5
19	1	MPNPNQ	70	95:5	99	3	LAIPPQ	73	77:23
20	1	MGKPDL	73	92:8	100	3	MKIVNQ	58	95:5
21	1	MKKPNQ	69	94:6	101	3	MPVVPS	58	96:4
22	1	MTLLNH	69	93:7	102	3	MILTNQ	58	94:6
23	1	VMTPTQ	76	83:17	103	3	MPPSNQ	55	96:4
24	1	MFAPNQ	66	96:4	104	3	MCYLNQ	54	95:5
25	1	MPLPNF	67	91:9	105	3	MRLPNQ	54	95:5
26	1	MSYTNA	61	93:7	106	3	MLATNQ	52	96:4
27	1	MKRPGQ	58	94:6	107	3	MQLPDV	52	95:5
28	1	MIHSPA	53	90:10	108	3	MHIPNL	54	90:10
29	1	QTVDDQ	50	91:9	109	3	MMIVNQ	50	93:7
30	1	MIAHVQ	51	88:12	110	3	MPQTDQ	49	94:6
31	1	MPLPKR	49	92:8	111	3	MPTSEM	49	92:8
32	1	HDAPNA	45	82:18	112	3	VQFPPQ	52	82:18
33	1	NALTNF	52	68:32	113	3	MQWCAN	45	94:6
34	1	MPPPRQ	37	94:6	114	3	MVWAHA	46	93:7
35	1	KVLPNV	46	73:27	115	3	LAFPNQ	57	74:26
36	1	VPLTNL	38	87:13	116	3	MERRNR	43	95:5
37	1	FPNPNQ	42	73:27	117	3	LQLTNL	55	71:29
38	1	FRAPDP	41	72:28	118	3	MPVTSL	41	92:8
39	1	YPLPVQ	37	76:24	119	3	IPLANQ	46	80:20
40	1	FLLPDQ	38	74:26	120	3	VQFPPQ	43	83:17
41	1	FIRLNQ	38	69:31	121	3	NKLPEG	46	76:24
42	1	FIRLNQ	35	67:33	122	3	QPNPNA	40	86:14
43	2	MPLVSQ	101	95:5	123	3	NVIPNQ	41	79:21
44	2	MVQYNE	98	97:3	124	3	FMLPSQ	46	70:30
45	2	MELVYM	99	95:5	125	3	FILHNQ	35	70:30
46	2	MQIPNQ	96	96:4	126	3	YPLTNQ	26	72:28
47	2	MVALDQ	88	95:5	127	3	FIRLNQ	24	69:31
48	2	MQVANQ	86	96:4	128	4	MAFPDQ	121	96:4
49	2	MVCMNQ	84	96:4	129	4	MALPDM	110	96:4
50	2	ALLPER	116	67:33	130	4	MLLSDA	108	96:4
51	2	SPIPAM	91	85:15	131	4	MPIPNQ	106	96:4
52	2	QPVPNF	89	86:14	132	4	MEVPFQ	105	96:4
53	2	MECTDQ	77	96:4	133	4	MESANQ	105	97:3
54	2	MPTPNH	77	95:5	134	4	MPPANQ	104	96:4
55	2	MTLTNT	76	96:4	135	4	MQQAGR	103	95:5
56	2	MALPDM	74	96:4	136	4	MRLTNQ	102	96:4
57	2	MMVTNQ	71	96:4	137	4	MPNPNQ	101	96:4
58	2	MCQPYL	71	95:5	138	4	MIVTNQ	101	96:4
59	2	MALPNM	70	96:4	139	4	MAIPPQ	100	97:3
60	2	LSPYDQ	78	80:20	140	4	MSLPAQ	101	96:4
61	2	MPLVSQ	64	94:6	141	4	ILEPNL	99	97:3
62	2	MPSWNQ	64	95:5	142	4	MALPDM	98	96:4
63	2	MMLTNQ	62	96:4	143	4	MQFAAQ	98	96:4
64	2	VSPPTQ	70	84:16	144	4	MGLTQM	96	96:4
65	2	MHLDPQ	62	94:6	145	4	MQQAGR	95	95:5
66	2	MPRKDA	61	95:5	146	4	MVFHEP	89	96:4
67	2	MVLNST	58	95:5	147	4	MPFPNQ	86	96:4
68	2	MDAPKH	54	95:5	148	4	MKLTHQ	77	96:4
69	2	MLLPAC	52	91:9	149	4	MKKTNA	74	96:4
70	2	MPLPTK	47	90:10	150	4	MPLADF	76	92:8
71	2	MPLIAL	43	88:12	151	4	MFRAKQ	63	95:5
72	2	MRFAAQ	41	92:8	152	4	MLVPNQ	71	79:21
73	2	MFTKRQ	36	92:8	153	4	YWVPNQ	42	77:23
74	2	YPLPNQ	41	75:25	154	4	FNAINR	45	67:33
75	2	MACTDK	29	94:6	155	4	YGHLSQ	40	74:26
76	2	HQLPQM	34	80:20	156	4	FPCASQ	38	71:29
77	2	FPVAEL	37	67:33	157	4	MYLTNQ	41	66:34
78	2	RSLPNQ	19	81:19	158	4	DCLVNQ	29	70:30
79	2	MALPNQ	14	92:8	159	4	MNFPNQ	32	58:42
80	3	MSETMQ	85	96:4	160	4	MPLNDF	7	55:45

Supplementary Table 3. Screening results of the MODIFY library of *Rma* cytochrome *c* for C–B bond formation reaction. The catalytic activity (i.e., yield) and enantioselectivity (i.e., enantiomeric ratio (e.r.)) of the variants in the MODIFY library were reported. For each plate, the variants were ranked according to the values of yield × major_enantiomer in descending order.

entry	plate	variant (75,99-103)	yield (%)	e.r.	entry	plate	variant (75,99-103)	yield (%)	e.r.
1	1	MVKPNP	53	99:1	81	3	MRWPWQ	39	99:1
2	1	MLLTAQ	51	98:2	82	3	VQFPPQ	36	99:1
3	1	MELQNQ	44	99:1	83	3	LAFPNQ	33	98:2
4	1	MLYPPT	44	99:1	84	3	KPWPNY	33	99:1
5	1	MVYGDQ	44	98:2	85	3	LQIPNQ	33	99:1
6	1	MPEPNQ	42	>99.9:0.1	86	3	MQVPTQ	32	99:1
7	1	MGAANQ	40	99:1	87	3	MTVPNQ	32	97:3
8	1	SFLTNQ	40	96:4	88	3	MRLPNQ	30	99:1
9	1	MMLTDQ	39	98:2	89	3	MVWAHA	30	99:1
10	1	MRNPNQ	39	98:2	90	3	MPAEFQ	30	99.6:0.4
11	1	MQLVDQ	39	99:1	91	3	SNAPPT	30	98:2
12	1	MPNTNV	39	98:2	92	3	MPEPNQ	29	99:1
13	1	MGKPDL	38	97:3	93	3	MCYLNQ	29	99:1
14	1	MVAAPL	38	97:3	94	3	ALLGQT	28	99:1
15	1	MTLLNH	37	98:2	95	3	MLLSDA	27	99:1
16	1	MPIPDQ	36	98:2	96	3	MQWCAN	27	99:1
17	1	MALMNM	36	98:2	97	3	MSETMQ	27	99:1
18	1	MDEPPQ	36	98:2	98	3	SRFTDM	27	98:2
19	1	MKKPNQ	36	97:3	99	3	APIANQ	27	99:1
20	1	MFAPNQ	35	98:2	100	3	MLATNQ	27	97:3
21	1	MPLPNF	34	97:3	101	3	MPVTSL	26	99:1
22	1	MPIPGQ	33	95:5	102	3	QPNPNA	26	99:1
23	1	MPIPDQ	32	99:1	103	3	MMIVNQ	26	99:1
24	1	VMTPTQ	33	95:5	104	3	LAIPPQ	26	98:2
25	1	MPLPKR	30	99:1	105	3	MQLPDV	25	99:1
26	1	HDAPNA	31	97:3	106	3	MRFPDQ	25	99:1
27	1	MSYTNA	30	97:3	107	3	MHLRNN	25	99:1
28	1	MKRPGQ	27	97:3	108	3	MILTNQ	25	97:3
29	1	MPNPNQ	26	97:3	109	3	MAIPAQ	23	99:1
30	1	VPLTNL	26	95:5	110	3	LQLTNL	24	96:4
31	1	MPPPRQ	23	98:2	111	3	MIITNQ	23	98:2
32	1	MIAHVQ	21	97:3	112	3	MPPSNQ	22	99:1
33	1	MIHSPA	19	92:8	113	3	MPQPNQ	22	99:1
34	1	QTVDDQ	20	90:10	114	3	MPVVPS	22	99:1
35	1	KVLPNV	18	82:18	115	3	IPLANQ	19	99.8:0.2
36	1	FIRLNQ	19	68:32	116	3	NVIPNQ	19	96:4
37	1	NALTNF	16	80:20	117	3	MPQTDQ	19	99:1
38	1	FLLPDQ	16	76:24	118	3	MPTSEM	17	99:1
39	1	FIRLNQ	17	70:30	119	3	NKLPEG	17	97:3
40	1	FPNPNQ	15	75:25	120	3	MERRNR	15	98:2
41	1	YPLPVQ	12	77:23	121	3	MKIVNQ	15	98:2
42	1	FRAPDP	11	75:25	122	3	MHIPNL	16	91:9
43	2	ALLPER	34	97:3	123	3	MPFPVQ	14	97:3
44	2	MQVANQ	33	99:1	124	3	YPLTNQ	14	89:11
45	2	MTLTNT	33	96:4	125	3	FILHNQ	9	84:16
46	2	MPSWNQ	29	98:2	126	3	FMLPSQ	8	88:12
47	2	SPIPAM	29	98:2	127	3	FIRLNQ	5	84:16
48	2	MLLPAC	29	99:1	128	4	MPIPNQ	32	99:1
49	2	MVLNST	29	99:1	129	4	MPNPNQ	31	99:1
50	2	MPLIAL	29	97:3	130	4	MRLTNQ	31	99:1
51	2	MCQPYL	28	98:2	131	4	MEVPFQ	30	99:1
52	2	MPTPNH	28	96:4	132	4	MPPANQ	29	99:1
53	2	MECTDQ	27	99:1	133	4	MLLSDA	29	99:1
54	2	MALPNM	26	99:1	134	4	MPFPNQ	28	99:1
55	2	MALPDM	26	98:2	135	4	MIVTNQ	27	99:1
56	2	MELVYM	26	96:4	136	4	MALPDM	26	99:1
57	2	MPLVSQ	25	99:1	137	4	MAFPDQ	25	99:1
58	2	MALPNQ	24	99:1	138	4	MKLTHQ	25	98:2
59	2	MDAPKH	24	98:2	139	4	MAIPPQ	24	97:3
60	2	MQIPNQ	23	99:1	140	4	MALPDM	24	99:1
61	2	MMLTNQ	23	99:1	141	4	MESANQ	23	99:1
62	2	LSPYDQ	24	97:3	142	4	MQQAGR	23	99.6:0.4
63	2	MPLVSQ	23	96:4	143	4	YWVPNQ	24	94:6
64	2	MVALDQ	22	99:1	144	4	ILEPNL	22	99:1
65	2	MVQYNE	22	95:5	145	4	MQQAGR	22	99:1
66	2	MRFAAQ	22	95:5	146	4	MPLADF	21	98:2
67	2	MMVTNQ	21	99:1	147	4	MKKTNA	18	99:1
68	2	MVCMNQ	21	97:3	148	4	MGLTQM	18	99:1
69	2	VSPPTQ	19	99:1	149	4	MQFAAQ	18	95:5
70	2	MPRKDA	19	99:1	150	4	MFRAKQ	16	95:5
71	2	MHLDPQ	18	98:2	151	4	DCLVNQ	17	87:13
72	2	MACTDK	17	99:1	152	4	MLVPNQ	15	94:6
73	2	QPVPNF	16	98:2	153	4	YGHLSQ	14	89:11
74	2	MFTKRQ	15	97:3	154	4	FPCASQ	12	79:21
75	2	YPLPNQ	14	92:8	155	4	MVFHEP	11	87:13
76	2	MPLPTK	12	98:2	156	4	MSLPAQ	9	94:6
77	2	HQLPQM	10	94:6	157	4	FNAINR	8	87:13
78	2	RSLPNQ	9	95:5	158	4	MYLTNQ	6	81:19
79	2	FPVAEL	7	85:15	159	4	MNFPNQ	5	75:25
80	3	VQFPPQ	42	99:1	160	4	MPLNDF	4	91:9

Supplementary Table 4. Screening results of the MODIFY library of *Rma* cytochrome *c* for C–Si bond formation reaction. The catalytic activity (i.e., yield) and enantioselectivity (i.e., enantiomeric ratio (e.r.)) of the variants in the MODIFY library were reported. For each plate, the variants were ranked according to the values of yield × major_enantiomer in descending order.

entry	plate	well	yield (%)	e.r.	entry	plate	well	yield (%)	e.r.	entry	plate	well	yield (%)	e.r.	entry	plate	well	yield (%)	e.r.
1	1	A2	27	71:29	93	2	A2	32	87:13	185	3	A2	23	60:40	277	4	A2	58	74:26
2	1	A3	71	76:24	94	2	A3	15	53:47	186	3	A3	51	83:17	278	4	A3	34	72:28
3	1	A4	68	77:23	95	2	A4	24	83:17	187	3	A4	65	76:24	279	4	A4	54	78:22
4	1	A5	50	77:23	96	2	A5	57	83:17	188	3	A5	16	51:49	280	4	A5	16	53:47
5	1	A6	29	68:32	97	2	A6	24	69:31	189	3	A6	15	54:46	281	4	A6	47	78:22
6	1	A7	7	55:45	98	2	A7	18	51:49	190	3	A7	63	94:6	282	4	A7	31	67:33
7	1	A8	31	62:38	99	2	A8	38	87:13	191	3	A8	30	64:36	283	4	A8	33	91:9
8	1	A9	55	91:9	100	2	A9	42	79:21	192	3	A9	22	53:47	284	4	A9	12	57:43
9	1	A10	62	81:19	101	2	A10	18	58:42	193	3	A10	24	53:47	285	4	A10	45	73:27
10	1	A11	51	79:21	102	2	A11	35	84:16	194	3	A11	19	52:48	286	4	A11	26	65:35
11	1	A12	30	72:28	103	2	A12	44	87:13	195	3	A12	8	53:47	287	4	A12	48	80:20
12	1	B1	59	94:6	104	2	B1	21	71:29	196	3	B1	29	62:38	288	4	B1	8	62:38
13	1	B3	53	82:18	105	2	B3	51	92:8	197	3	B3	26	80:20	289	4	B3	18	58:42
14	1	B4	42	81:19	106	2	B4	53	81:19	198	3	B4	47	91:9	290	4	B4	14	52:48
15	1	B5	21	59:41	107	2	B5	35	78:22	199	3	B5	50	91:9	291	4	B5	25	66:34
16	1	B6	13	60:40	108	2	B6	47	90:10	200	3	B6	46	72:28	292	4	B6	5	54:46
17	1	B7	37	88:12	109	2	B7	46	82:18	201	3	B7	63	69:31	293	4	B7	26	71:29
18	1	B8	29	67:33	110	2	B8	51	82:18	202	3	B8	25	64:36	294	4	B8	15	64:36
19	1	B9	20	71:29	111	2	B9	18	68:32	203	3	B9	55	84:16	295	4	B9	16	52:48
20	1	B10	39	88:12	112	2	B10	28	62:38	204	3	B10	0	50:50	296	4	B10	17	52:48
21	1	B11	79	87:13	113	2	B11	16	65:35	205	3	B11	38	70:30	297	4	B11	18	61:39
22	1	B12	0	50:50	114	2	B12	29	79:21	206	3	B12	21	64:36	298	4	B12	36	77:23
23	1	C1	20	63:37	115	2	C1	30	66:34	207	3	C1	43	78:22	299	4	C1	8	54:46
24	1	C2	11	51:49	116	2	C2	20	65:35	208	3	C2	41	81:19	300	4	C2	9	51:49
25	1	C4	7	50:50	117	2	C4	26	72:28	209	3	C4	8	50:50	301	4	C4	22	53:47
26	1	C5	62	85:15	118	2	C5	44	76:24	210	3	C5	28	88:12	302	4	C5	42	83:17
27	1	C6	9	52:48	119	2	C6	16	53:47	211	3	C6	7	51:49	303	4	C6	11	58:42
28	1	C7	68	95:5	120	2	C7	43	91:9	212	3	C7	25	61:39	304	4	C7	7	52:48
29	1	C8	31	75:25	121	2	C8	17	56:44	213	3	C8	10	67:33	305	4	C8	29	69:31
30	1	C9	46	70:30	122	2	C9	16	59:41	214	3	C9	24	85:15	306	4	C9	20	61:39
31	1	C10	28	78:22	123	2	C10	13	51:49	215	3	C10	17	60:40	307	4	C10	45	93:7
32	1	C11	32	81:19	124	2	C11	11	53:47	216	3	C11	17	59:41	308	4	C11	50	89:11
33	1	C12	41	80:20	125	2	C12	19	53:47	217	3	C12	30	70:30	309	4	C12	41	70:30
34	1	D1	15	52:48	126	2	D1	20	68:32	218	3	D1	17	61:39	310	4	D1	38	74:26
35	1	D2	18	63:37	127	2	D2	68	85:15	219	3	D2	53	93:7	311	4	D2	15	65:35
36	1	D3	9	52:48	128	2	D3	14	58:42	220	3	D3	28	86:14	312	4	D3	37	91:9
37	1	D5	9	50:50	129	2	D5	45	77:23	221	3	D5	13	66:34	313	4	D5	26	86:14
38	1	D6	22	71:29	130	2	D6	21	70:30	222	3	D6	17	58:42	314	4	D6	18	68:32
39	1	D7	28	83:17	131	2	D7	53	90:10	223	3	D7	9	54:46	315	4	D7	26	61:39
40	1	D8	14	50:50	132	2	D8	31	69:31	224	3	D8	34	80:20	316	4	D8	28	59:41
41	1	D9	49	74:26	133	2	D9	46	72:28	225	3	D9	9	53:47	317	4	D9	38	70:30
42	1	D10	16	51:49	134	2	D10	42	67:33	226	3	D10	46	76:24	318	4	D10	33	87:13
43	1	D11	39	81:19	135	2	D11	46	74:26	227	3	D11	39	90:10	319	4	D11	42	70:30
44	1	D12	28	53:47	136	2	D12	22	76:24	228	3	D12	48	92:8	320	4	D12	57	95:5
45	1	E1	20	61:39	137	2	E1	23	53:47	229	3	E1	38	72:28	321	4	E1	39	70:30
46	1	E2	16	60:40	138	2	E2	27	63:37	230	3	E2	23	56:44	322	4	E2	64	94:6
47	1	E3	23	64:36	139	2	E3	8	51:49	231	3	E3	32	78:22	323	4	E3	19	59:41
48	1	E4	14	65:35	140	2	E4	21	71:29	232	3	E4	20	53:47	324	4	E4	38	56:44
49	1	E5	10	51:49	141	2	E5	29	76:24	233	3	E5	9	51:49	325	4	E5	50	76:24
50	1	E6	44	86:14	142	2	E6	39	90:10	234	3	E6	45	92:8	326	4	E6	58	93:7
51	1	E7	29	59:41	143	2	E7	32	65:35	235	3	E7	0	50:50	327	4	E7	35	76:24
52	1	E8	47	92:8	144	2	E8	63	93:7	236	3	E8	29	85:15	328	4	E8	30	66:34
53	1	E9	20	59:41	145	2	E9	32	64:36	237	3	E9	24	86:14	329	4	E9	18	62:38
54	1	E10	40	78:22	146	2	E10	0	50:50	238	3	E10	10	55:45	330	4	E10	31	63:37
55	1	E11	38	63:37	147	2	E11	30	54:46	239	3	E11	25	77:23	331	4	E11	22	64:36
56	1	E12	34	87:13	148	2	E12	20	62:38	240	3	E12	18	53:47	332	4	E12	45	71:29
57	1	F1	20	59:41	149	2	F1	50	79:21	241	3	F1	19	58:42	333	4	F1	50	71:29
58	1	F2	25	77:23	150	2	F2	30	55:45	242	3	F2	48	69:31	334	4	F2	11	51:49
59	1	F3	10	55:45	151	2	F3	50	76:24	243	3	F3	49	82:18	335	4	F3	27	68:32
60	1	F4	15	51:49	152	2	F4	0	50:50	244	3	F4	46	90:10	336	4	F4	42	72:28
61	1	F5	13	56:44	153	2	F5	54	80:20	245	3	F5	10	63:37	337	4	F5	35	67:33
62	1	F6	25	74:26	154	2	F6	54	92:8	246	3	F6	30	79:21	338	4	F6	63	75:25
63	1	F7	20	63:37	155	2	F7	37	70:30	247	3	F7	19	81:19	339	4	F7	32	69:31
64	1	F8	15	70:30	156	2	F8	43	91:9	248	3	F8	13	52:48	340	4	F8	34	63:37
65	1	F9	9	51:49	157	2	F9	64	75:25	249	3	F9	51	94:6	341	4	F9	41	60:40
66	1	F10	16	56:44	158	2	F10	55	69:31	250	3	F10	12	52:48	342	4	F10	26	54:46
67	1	F11	23	62:38	159	2	F11	12	51:49	251	3	F11	23	59:41	343	4	F11	72	84:16
68	1	F12	8	51:49	160	2	F12	35	66:34	252	3	F12	42	91:9	344	4	F12	30	62:38
69	1	G1	58	77:23	161	2	G1	37	90:10	253	3	G1	40	81:19	345	4	G1	72	84:16
70	1	G2	21	61:39	162	2	G2	34	73:27	254	3	G2	5	50:50	346	4	G2	43	78:22
71	1	G3	8	53:47	163	2	G3	8	50:50	255	3	G3	28	83:17	347	4	G3	66	76:24
72	1	G4	13	52:48	164	2	G4	34	65:35	256	3	G4	5	51:49	348	4	G4	45	63:37
73	1	G5	12	51:49	165	2	G5	18	53:47	257	3	G5	34	90:10	349	4	G5	24	57:43
74	1	G6	24	68:32	166	2	G6	23	64:36	258	3	G6	39	79:21	350	4	G6	23	57:43
75	1	G7	28	73:27	167	2	G7	36	85:15	259	3	G7	0	50:50	351	4	G7	8	54:46
76	1	G8	55	73:27	168	2	G8	38	70:30	260	3	G8	47	93:7	352	4	G8	67	82:18
77	1	G9	14	52:48	169	2	G9	38	75:25	261	3	G9	26	83:17	353	4	G9	35	78:22
78	1	G10	47	78:22	170	2	G10	40	80:20	262	3	G10	24	84:16	354	4	G10	30	66:34
79	1	G11	59	79:21	171	2	G11	43	71:29	263	3	G11	40	91:9	355	4	G11	49	91:9
80	1	G12	66	83:17	172	2	G12	34	88:12	264	3	G12	9	54:46	356	4	G12	17	61:39
81	1	H1	61	95:5	173	2	H1	29	90:10	265	3	H1	44	85:15	357	4	H1	18	53:47
82	1	H2	39	91:9	174	2	H2	45	76:24	266	3	H2	47	79:21	358	4	H2	35	73:27
83	1	H3	51	76:24	175	2	H3	26	69:31	267	3	H3	21	63:37	359	4	H3	22	59:41
84	1	H4	12	64:36	176	2	H4	26	64:36	268	3	H4	5	50:50	360	4	H4	40	78:22
85	1	H5	52	93:7	177	2	H5	31	90:10	269	3	H5	10	54:46	361	4	H5	39	84:16
86	1	H6	34	89:11	178	2	H6	23	72:28	270	3	H6	34	94:6	362	4	H6	52	81:19
87	1	H7	12	62:38	179	2	H7	37	66:34	271	3	H7	11	63:37	363	4	H7	17	61:39
88	1	H8	55	86:14	180	2	H8	20	63:37	272	3	H8	16	66:34	364	4	H8	20	64:36
89	1	H9	29	83:17	181	2	H9	34	68:32	273	3	H9	24	76:24	365	4	H9	33	90:10
90	1	H10	36	71:29	182	2	H10	42	60:40	274	3	H10	6	52:48	366	4	H10	18	58:42
91	1	H11	10	50:50	183	2	H11	15	66:34	275	3	H11	11	59:41	367	4	H11	20	61:39
92	1	H12	18	63:37	184	2	H12	7	64:36	276	3	H12	12	70:30	368	4	H12	37	60:40

Supplementary Table 5. Screening results of the NNK library of *Rma* cytochrome *c* for C–B bond formation reaction. The catalytic activity (i.e., yield) and enantioselectivity (i.e., enantiomeric ratio (e.r.)) of the variants in the NNK library were reported.

entry	plate	well	yield (%)	e.r.	entry	plate	well	yield (%)	e.r.	entry	plate	well	yield (%)	e.r.	entry	plate	well	yield (%)	e.r.
1	1	A2	25	96:4	93	2	A2	6	95:5	185	3	A2	7	90:10	277	4	A2	25	98:2
2	1	A3	33	98:2	94	2	A3	3	89:11	186	3	A3	33	99:1	278	4	A3	20	96:4
3	1	A4	39	98:2	95	2	A4	11	98:2	187	3	A4	24	98:2	279	4	A4	30	99:1
4	1	A5	19	98:2	96	2	A5	13	97:3	188	3	A5	5	80:20	280	4	A5	6	75:25
5	1	A6	4	95:5	97	2	A6	12	98:2	189	3	A6	6	77:23	281	4	A6	21	96:4
6	1	A7	8	67:33	98	2	A7	5	68:32	190	3	A7	30	99:1	282	4	A7	13	83:17
7	1	A8	14	87:13	99	2	A8	13	98:2	191	3	A8	10	88:12	283	4	A8	25	99:1
8	1	A9	15	98:2	100	2	A9	16	98:2	192	3	A9	7	75:25	284	4	A9	5	85:15
9	1	A10	28	98:2	101	2	A10	4	76:24	193	3	A10	7	75:25	285	4	A10	25	98:2
10	1	A11	29	98:2	102	2	A11	7	96:4	194	3	A11	7	76:24	286	4	A11	11	86:14
11	1	A12	14	95:5	103	2	A12	8	96:4	195	3	A12	7	73:27	287	4	A12	21	98:2
12	1	B1	33	100:0	104	2	B1	9	96:4	196	3	B1	5	82:18	288	4	B1	7	96:4
13	1	B3	23	98:2	105	2	B3	8	98:2	197	3	B3	12	97:3	289	4	B3	11	97:3
14	1	B4	25	98:2	106	2	B4	15	98:2	198	3	B4	18	99:1	290	4	B4	7	68:32
15	1	B5	12	84:16	107	2	B5	14	96:4	199	3	B5	16	98:2	291	4	B5	16	96:4
16	1	B6	8	86:14	108	2	B6	12	98:2	200	3	B6	28	98:2	292	4	B6	6	71:29
17	1	B7	21	98:2	109	2	B7	16	98:2	201	3	B7	26	98:2	293	4	B7	24	98:2
18	1	B8	15	85:15	110	2	B8	17	98:2	202	3	B8	14	89:11	294	4	B8	10	91:9
19	1	B9	11	95:5	111	2	B9	6	91:9	203	3	B9	26	98:2	295	4	B9	6	69:31
20	1	B10	13	97:3	112	2	B10	7	91:9	204	3	B10	6	100:0	296	4	B10	7	68:32
21	1	B11	5	100:0	113	2	B11	3	84:16	205	3	B11	18	88:12	297	4	B11	9	87:13
22	1	B12	5	99:1	114	2	B12	8	95:5	206	3	B12	8	82:18	298	4	B12	21	98:2
23	1	C1	8	87:13	115	2	C1	12	97:3	207	3	C1	21	97:3	299	4	C1	4	82:18
24	1	C2	7	68:32	116	2	C2	6	85:15	208	3	C2	16	97:3	300	4	C2	6	80:20
25	1	C4	5	83:17	117	2	C4	12	97:3	209	3	C4	7	72:28	301	4	C4	7	67:33
26	1	C5	26	98:2	118	2	C5	18	96:4	210	3	C5	25	99:1	302	4	C5	30	98:2
27	1	C6	5	75:25	119	2	C6	6	63:37	211	3	C6	8	70:30	303	4	C6	9	89:11
28	1	C7	33	100:0	120	2	C7	11	99:1	212	3	C7	16	84:16	304	4	C7	7	70:30
29	1	C8	5	100:0	121	2	C8	6	72:28	213	3	C8	21	98:2	305	4	C8	17	94:6
30	1	C9	21	98:2	122	2	C9	4	77:23	214	3	C9	37	99:1	306	4	C9	14	85:15
31	1	C10	17	97:3	123	2	C10	4	72:28	215	3	C10	13	88:12	307	4	C10	22	99:1
32	1	C11	16	98:2	124	2	C11	4	84:16	216	3	C11	7	74:26	308	4	C11	25	99:1
33	1	C12	17	96:4	125	2	C12	4	71:29	217	3	C12	10	88:12	309	4	C12	14	87:13
34	1	D1	6	73:27	126	2	D1	12	91:9	218	3	D1	8	89:11	310	4	D1	20	97:3
35	1	D2	7	79:21	127	2	D2	8	91:9	219	3	D2	23	100:0	311	4	D2	16	97:3
36	1	D3	6	72:28	128	2	D3	11	97:3	220	3	D3	17	99:1	312	4	D3	33	100:0
37	1	D5	8	69:31	129	2	D5	17	98:2	221	3	D5	8	89:11	313	4	D5	9	93:7
38	1	D6	6	95:5	130	2	D6	6	90:10	222	3	D6	8	75:25	314	4	D6	15	95:5
39	1	D7	18	98:2	131	2	D7	4	88:12	223	3	D7	9	83:17	315	4	D7	7	74:26
40	1	D8	7	66:34	132	2	D8	8	87:13	224	3	D8	21	98:2	316	4	D8	10	79:21
41	1	D9	20	97:3	133	2	D9	4	99:1	225	3	D9	5	84:16	317	4	D9	14	87:13
42	1	D10	8	67:33	134	2	D10	12	82:18	226	3	D10	36	99:1	318	4	D10	13	99:1
43	1	D11	20	97:3	135	2	D11	15	98:2	227	3	D11	21	99:1	319	4	D11	17	85:15
44	1	D12	7	68:32	136	2	D12	10	97:3	228	3	D12	10	95:5	320	4	D12	25	99:1
45	1	E1	20	97:3	137	2	E1	5	67:33	229	3	E1	9	89:11	321	4	E1	8	86:14
46	1	E2	15	89:11	138	2	E2	5	71:29	230	3	E2	8	75:25	322	4	E2	30	100:0
47	1	E3	12	86:14	139	2	E3	4	74:26	231	3	E3	9	97:3	323	4	E3	6	74:26
48	1	E4	11	90:10	140	2	E4	8	96:4	232	3	E4	8	69:31	324	4	E4	8	67:33
49	1	E5	7	68:32	141	2	E5	11	95:5	233	3	E5	5	75:25	325	4	E5	20	98:2
50	1	E6	21	98:2	142	2	E6	13	99:1	234	3	E6	14	97:3	326	4	E6	32	100:0
51	1	E7	14	84:16	143	2	E7	9	79:21	235	3	E7	6	100:0	327	4	E7	38	99:1
52	1	E8	24	99:1	144	2	E8	7	96:4	236	3	E8	6	97:3	328	4	E8	11	86:14
53	1	E9	8	84:16	145	2	E9	8	84:16	237	3	E9	12	96:4	329	4	E9	7	87:13
54	1	E10	16	97:3	146	2	E10	4	100:0	238	3	E10	8	90:10	330	4	E10	9	82:18
55	1	E11	9	84:16	147	2	E11	4	68:32	239	3	E11	13	95:5	331	4	E11	9	89:11
56	1	E12	11	98:2	148	2	E12	5	75:25	240	3	E12	8	70:30	332	4	E12	23	98:2
57	1	F1	12	67:33	149	2	F1	12	97:3	241	3	F1	5	80:20	333	4	F1	17	96:4
58	1	F2	21	99:1	150	2	F2	6	68:32	242	3	F2	24	98:2	334	4	F2	5	81:19
59	1	F3	15	84:16	151	2	F3	12	96:4	243	3	F3	26	98:2	335	4	F3	4	93:7
60	1	F4	6	70:30	152	2	F4	3	100:0	244	3	F4	17	98:2	336	4	F4	18	97:3
61	1	F5	6	76:24	153	2	F5	15	95:5	245	3	F5	7	83:17	337	4	F5	14	91:9
62	1	F6	29	98:2	154	2	F6	8	98:2	246	3	F6	21	98:2	338	4	F6	27	99:1
63	1	F7	13	88:12	155	2	F7	10	83:17	247	3	F7	22	99:1	339	4	F7	13	91:9
64	1	F8	12	97:3	156	2	F8	10	97:3	248	3	F8	7	72:28	340	4	F8	14	87:13
65	1	F9	8	66:34	157	2	F9	8	98:2	249	3	F9	28	99:1	341	4	F9	10	82:18
66	1	F10	13	79:21	158	2	F10	11	97:3	250	3	F10	8	69:31	342	4	F10	8	72:28
67	1	F11	17	94:6	159	2	F11	4	69:31	251	3	F11	13	83:17	343	4	F11	30	98:2
68	1	F12	6	67:33	160	2	F12	8	87:13	252	3	F12	17	98:2	344	4	F12	8	86:14
69	1	G1	27	97:3	161	2	G1	12	99:1	253	3	G1	27	99:1	345	4	G1	21	98:2
70	1	G2	13	85:15	162	2	G2	11	83:17	254	3	G2	6	94:6	346	4	G2	16	96:4
71	1	G3	5	74:26	163	2	G3	4	74:26	255	3	G3	15	98:2	347	4	G3	25	98:2
72	1	G4	5	71:29	164	2	G4	7	85:15	256	3	G4	4	82:18	348	4	G4	7	73:27
73	1	G5	8	75:25	165	2	G5	4	71:29	257	3	G5	6	94:6	349	4	G5	8	73:27
74	1	G6	12	86:14	166	2	G6	8	85:15	258	3	G6	26	97:3	350	4	G6	6	75:25
75	1	G7	23	96:4	167	2	G7	6	88:12	259	3	G7	6	100:0	351	4	G7	8	68:32
76	1	G8	15	96:4	168	2	G8	12	85:15	260	3	G8	16	98:2	352	4	G8	17	97:3
77	1	G9	6	71:29	169	2	G9	13	96:4	261	3	G9	8	90:10	353	4	G9	32	98:2
78	1	G10	15	97:3	170	2	G10	17	97:3	262	3	G10	14	97:3	354	4	G10	22	98:2
79	1	G11	22	97:3	171	2	G11	10	84:16	263	3	G11	14	97:3	355	4	G11	10	96:4
80	1	G12	28	99:1	172	2	G12	7	97:3	264	3	G12	36	98:2	356	4	G12	6	84:16
81	1	H1	28	99:1	173	2	H1	5	94:6	265	3	H1	19	97:3	357	4	H1	6	68:32
82	1	H2	17	98:2	174	2	H2	12	98:2	266	3	H2	20	98:2	358	4	H2	14	97:3
83	1	H3	19	98:2	175	2	H3	8	96:4	267	3	H3	11	81:19	359	4	H3	4	82:18
84	1	H4	12	84:16	176	2	H4	5	84:16	268	3	H4	4	81:19	360	4	H4	27	99:1
85	1	H5	29	100:0	177	2	H5	8	99:1	269	3	H5	5	78:22	361	4	H5	12	98:2
86	1	H6	23	100:0	178	2	H6	7	91:9	270	3	H6	9	96:4	362	4	H6	24	98:2
87	1	H7	5	87:13	179	2	H7	15	97:3	271	3	H7	10	87:13	363	4	H7	9	90:10
88	1	H8	24	99:1	180	2	H8	6	85:15	272	3	H8	7	85:15	364	4	H8	6	89:11
89	1	H9	4	100:0	181	2	H9	11	97:3	273	3	H9	29	98:2	365	4	H9	4	100:0
90	1	H10	9	86:14	182	2	H10	13	97:3	274	3	H10	5	81:19	366	4	H10	7	82:18
91	1	H11	5	75:25	183	2	H11	4	86:14	275	3	H11	4	86:14	367	4	H11	8	85:15
92	1	H12	15	96:4	184	2	H12	4	88:12	276	3	H12	9	87:13	368	4	H12	22	98:2

Supplementary Table 6. Screening results of the NNK library of *Rma* cytochrome *c* for C–Si bond formation reaction. The catalytic activity (i.e., yield) and enantioselectivity (i.e., enantiomeric ratio (e.r.)) of the variants in the NNK library were reported.

Component	Final concentration	Per 25 µL reaction
5x KAPA HiFi Fidelity Buffer	1x	5.0 µL
10 mM dNTP Mix	0.3 mM dNTP	0.75 µL
10 µM Forward Primer	0.3 µM	0.75 µL
10 µM Reverse Primer	0.3 µM	0.75 µL
Twist Oligo Pool (20 ng/µL)	0.4 ng/µL	0.5 µL
KAPA HiFi HotStart DNA Polymerase (1 U/µL)	0.5 U/reaction	0.5 µL
PCR grade water	-	16.75 µL

Supplementary Table 7. PCR reaction components.

Cycling Step	Temperature	Duration
Initialization denaturation	3 min at 95 °C	1x
Denaturation	20 sec at 98 °C	12 cycles
Annealing	15 sec at 52 °C	
Extension	15 sec at 72 °C	
Final Extension	1 min at 72 °C	1x

Supplementary Table 8. PCR reaction conditions.

References

1 Notin, P. *et al.* Tranception: protein fitness prediction with autoregressive transformers and inference-time retrieval. In *International Conference on Machine Learning*, 16990–17017 (PMLR, 2022).

2 Hopf, T. A. *et al.* Mutation effects predicted from sequence co-variation. *Nat. Biotechnol.* **35**, 128–135 (2017).

3 Notin, P. *et al.* Proteingym: Large-scale benchmarks for protein design and fitness prediction. *bioRxiv* 2023–12 (2023).

4 Wu, N. C., Dai, L., Olson, C. A., Lloyd-Smith, J. O. & Sun, R. Adaptation in protein fitness landscapes is facilitated by indirect paths. *Elife* **5**, e16965 (2016).

5 Chen, Y. *et al.* Deep mutational scanning of an oxygen-independent fluorescent protein creilov for comprehensive profiling of mutational and epistatic effects. *ACS Synth. Biol.* **12**, 1461–1473 (2023).

6 Mukherjee, A. & Schroeder, C. M. Flavin-based fluorescent proteins: emerging paradigms in biological imaging. *Curr. Opin. Biotechnol.* **31**, 16–23 (2015).

7 Ding, D. *et al.* Co-evolution of interacting proteins through non-contacting and non-specific mutations. *Nat. Ecol. Evol.* **6**, 590–603 (2022).

8 Zhu, D. *et al.* Optimal trade-off control in machine learning–based library design, with application to adeno-associated virus (aav) for gene therapy. *Sci. Adv.* **10**, eadj3786 (2024).

9 Hopf, T. A. *et al.* The evcouplings python framework for coevolutionary sequence analysis. *Bioinformatics* **35**, 1582–1584 (2019).

10 Frazer, J. *et al.* Disease variant prediction with deep generative models of evolutionary data. *Nature* **599**, 91–95 (2021).

11 Rao, R. M. *et al.* MSA transformer. In *International Conference on Machine Learning*, 8844–8856 (PMLR, 2021).

12 Meier, J. *et al.* Language models enable zero-shot prediction of the effects of mutations on protein function. *Advances in Neural Information Processing Systems* **34**, 29287–29303 (2021).

13 Steinegger, M. *et al.* Hh-suite3 for fast remote homology detection and deep protein annotation. *BMC Bioinf.* **20**, 1–15 (2019).

14 Lin, Z. *et al.* Evolutionary-scale prediction of atomic-level protein structure with a language model. *Science* **379**, 1123–1130 (2023).

15 Schymkowitz, J. *et al.* The FoldX web server: an online force field. *Nucleic Acids Res.* **33**, W382–W388 (2005).

16 Jumper, J. *et al.* Highly accurate protein structure prediction with alphafold. *Nature* **596**, 583–589 (2021).

17 Olson, C. A., Wu, N. C. & Sun, R. A comprehensive biophysical description of pairwise epistasis throughout an entire protein domain. *Curr Biol* **24**, 2643–2651 (2014).

18 Protabit. `https://triad.protabit.com/`. [Online; accessed March 4, 2024].

19 Alford, R. F. *et al.* The rosetta all-atom energy function for macromolecular modeling and design. *J. Chem. Theory Comput.* **13**, 3031–3048 (2017).

20 Yang, J. *et al.* Decoil: Optimization of degenerate codon libraries for machine learning-assisted protein engineering. *ACS Synth. Biol.* **12**, 2444–2454 (2023).

21 Kan, S. J., Huang, X., Gumulya, Y., Chen, K. & Arnold, F. H. Genetically programmed chiral organoborane synthesis. *Nature* **552**, 132–136 (2017).

22 Kan, S. J., Lewis, R. D., Chen, K. & Arnold, F. H. Directed evolution of cytochrome c for carbon–silicon bond formation: Bringing silicon to life. *Science* **354**, 1048–1051 (2016).

23 Gibson, D. G. *et al.* Enzymatic assembly of dna molecules up to several hundred kilobases. *Nat. Methods* **6**, 343–345 (2009).

24 Berry, E. A. & Trumpower, B. L. Simultaneous determination of hemes a, b, and c from pyridine hemochrome spectra. *Anal. Biochem.* **161**, 1–15 (1987).

25 Barr, I. & Guo, F. Pyridine hemochromagen assay for determining the concentration of heme in purified protein solutions. *Bio-Protoc.* **5**, e1594–e1594 (2015).

26 Fiser, A. & Sali, A. ModLoop: automated modeling of loops in protein structures. *Bioinformatics* **19**, 2500–2501 (2003).

27 Schrödinger, LLC. The PyMOL molecular graphics system, version 1.8 (2015).

28 Lee, C., Yang, W. & Parr, R. G. Development of the colle-salvetti correlation-energy formula into a functional of the electron density. *Phys. Rev. B* **37**, 785–789 (1988).

29 Becke, A. D. Density-functional thermochemistry. III. The role of exact exchange. *J. Chem. Phys.* **98**, 5648–5652 (1993).

30 Frisch, M. J. *et al.* Gaussian 16 Revision C.01 (2016). Gaussian Inc. Wallingford CT.

31 Morris, G. M. *et al.* Autodock4 and autodocktools4: Automated docking with selective receptor flexibility. *J. Comput. Chem.* **30**, 2785–2791 (2009).

32 Salomon-Ferrer, R., Götz, A. W., Poole, D., Le Grand, S. & Walker, R. C. Routine microsecond molecular dynamics simulations with amber on gpus. 2. explicit solvent particle mesh ewald. *J. Chem. Theory Comput.* **9**, 3878–3888 (2013).

33 Case, D. *et al.* Amber 20 (2020). University of California, San Francisco, CA.

34 Maier, J. A. *et al.* ff14sb: Improving the accuracy of protein side chain and backbone parameters from ff99sb. *J. Chem. Theory Comput.* **11**, 3696–3713 (2015).

35 He, X., Man, V. H., Yang, W., Lee, T.-S. & Wang, J. A fast and high-quality charge model for the next generation general AMBER force field. *J. Chem. Phys.* **153**, 114502 (2020).

36 Li, P. & Merz, K. M. J. Mcpb.py: A python based metal center parameter builder. *J. Chem. Inf. Model.* **56**, 599–604 (2016).

37 Singh, U. C. & Kollman, P. A. An approach to computing electrostatic charges for molecules. *J. Comput. Chem.* **5**, 129–145 (1984).

38 Besler, B. H., Merz Jr., K. M. & Kollman, P. A. Atomic charges derived from semiempirical methods. *J. Comput. Chem.* **11**, 431–439 (1990).

39 Bayly, C. I., Cieplak, P., Cornell, W. & Kollman, P. A. A well-behaved electrostatic potential based method using charge restraints for deriving atomic charges: the resp model. *J. Phys. Chem.* **97**, 10269–10280 (1993).

40 Garcia-Borràs, M. *et al.* Origin and control of chemoselectivity in cytochrome c catalyzed carbene transfer into si–h and n–h bonds. *J. Am. Chem. Soc* **143**, 7114–7123 (2021).

41 Anandakrishnan, R., Aguilar, B. & Onufriev, A. V. H++ 3.0: automating pK prediction and the preparation of biomolecular structures for atomistic molecular modeling and simulations. *Nucleic Acids Res.* **40**, W537–W541 (2012).

42 Jorgensen, W. L., Chandrasekhar, J., Madura, J. D., Impey, R. W. & Klein, M. L. Comparison of simple potential functions for simulating liquid water. *J. Chem. Phys.* **79**, 926–935 (1983).

43 Ryckaert, J.-P., Ciccotti, G. & Berendsen, H. J. Numerical integration of the cartesian equations of motion of a system with constraints: molecular dynamics of n-alkanes. *J. Comput. Phys.* **23**, 327–341 (1977).

44 Darden, T., York, D. & Pedersen, L. Particle mesh Ewald: An $N \cdot \log(N)$ method for Ewald sums in large systems. *J. Chem. Phys.* **98**, 10089–10092 (1993).

45 Knorrscheidt, A. *et al.* Accessing chemo- and regioselective benzylic and aromatic oxidations by protein engineering of an unspecific peroxygenase. *ACS Catal.* **11**, 7327–7338 (2021).

46 Roe, D. R. & Cheatham, T. E. I. Ptraj and cpptraj: Software for processing and analysis of molecular dynamics trajectory data. *J. Chem. Theory Comput.* **9**, 3084–3095 (2013).

47 Sun, Z., Liu, Q., Qu, G., Feng, Y. & Reetz, M. T. Utility of b-factors in protein science: Interpreting rigidity, flexibility, and internal motion and engineering thermostability. *Chem. Rev.* **119**, 1626–1665 (2019).
