# Supplementary material for: Machine learning-guided co-optimization of fitness and diversity facilitates combinatorial library design in enzyme engineering
Source: Nat Commun. 2024 Jul 29;15:6392. doi: 10.1038/s41467-024-50698-y (PMC11289365; doi:10.1038/s41467-024-50698-y)
Supplement: Supplementary file 3 — Description of Additional Supplementary Files [file 41467_2024_50698_MOESM3_ESM.pdf]

Description of Additional Supplementary Files

File Name: Supplementary Data 1

Description: Oligo pool sequences of 1,000 *Rma* cytochrome *c* variants from the MODIFY-informed library.
