# Supplementary material for: Machine learning-guided co-optimization of fitness and diversity facilitates combinatorial library design in enzyme engineering
Source: Nat Commun. 2024 Jul 29;15:6392. doi: 10.1038/s41467-024-50698-y (PMC11289365; doi:10.1038/s41467-024-50698-y)
Supplement: Supplementary file 5 — Reporting Summary [file 41467_2024_50698_MOESM5_ESM.pdf]

nature portfolio

Corresponding author(s): Peng Liu, Yang Yang, Yunan Luo

Last updated by author(s): Jul 12, 2024

# Reporting Summary

Nature Portfolio wishes to improve the reproducibility of the work that we publish. This form provides structure for consistency and transparency in reporting. For further information on Nature Portfolio policies, see our Editorial Policies and the Editorial Policy Checklist.

## Statistics

For all statistical analyses, confirm that the following items are present in the figure legend, table legend, main text, or Methods section.

| n/a | Confirmed | |
|---|---|---|
| ☐ | ☒ | The exact sample size ($n$) for each experimental group/condition, given as a discrete number and unit of measurement |
| ☐ | ☒ | A statement on whether measurements were taken from distinct samples or whether the same sample was measured repeatedly |
| ☒ | ☐ | The statistical test(s) used AND whether they are one- or two-sided<br>*Only common tests should be described solely by name; describe more complex techniques in the Methods section.* |
| ☒ | ☐ | A description of all covariates tested |
| ☒ | ☐ | A description of any assumptions or corrections, such as tests of normality and adjustment for multiple comparisons |
| ☐ | ☒ | A full description of the statistical parameters including central tendency (e.g. means) or other basic estimates (e.g. regression coefficient) AND variation (e.g. standard deviation) or associated estimates of uncertainty (e.g. confidence intervals) |
| ☒ | ☐ | For null hypothesis testing, the test statistic (e.g. $F$, $t$, $r$) with confidence intervals, effect sizes, degrees of freedom and $P$ value noted<br>*Give P values as exact values whenever suitable.* |
| ☒ | ☐ | For Bayesian analysis, information on the choice of priors and Markov chain Monte Carlo settings |
| ☒ | ☐ | For hierarchical and complex designs, identification of the appropriate level for tests and full reporting of outcomes |
| ☐ | ☒ | Estimates of effect sizes (e.g. Cohen's $d$, Pearson's $r$), indicating how they were calculated |

*Our web collection on statistics for biologists contains articles on many of the points above.*

## Software and code

Policy information about availability of computer code

| | |
|---|---|
| Data collection | All experimental data were obtained through testing using the Shimadzu LC-2030C Plus and LC-2040C 3D Plus. The data were integrated using LabSolutions version 5.98 and 5.101, then copied to Excel for summary. |
| Data analysis | The source code of MODIFY is available at https://github.com/luo-group/MODIFY and has been deposited to Zenodo at https://doi.org/10.5281/zenodo.12715542. MODIFY was built on Python 3.9, PyTorch 2.0.0, Numpy 1.23.1, Pandas 1.4.4, Matplotlib 3.5.3, Logomaker 0.8, Seaborn 0.12.2, Biotite 0.38.0, Biopython 1.76. ESM-1v (https://github.com/facebookresearch/esm), ESM-2 (https://github.com/facebookresearch/esm), EVmutation (https://github.com/debbiemarkslab/EVcouplings), EVE (https://github.com/OATML-Markslab/EVE), and MSA Transformer (https://github.com/facebookresearch/esm) were used for zero-shot protein fitness prediction. ESMFold (https://github.com/facebookresearch/esm#esmfold) and FoldX5 (https://foldxsuite.crg.eu/) were used for structured-based filtering. For MD analysis, the following softwares were used: Amber 20, AutoDock 4.2, Gaussian 16, and Open-source PyMOL 3.0. |

For manuscripts utilizing custom algorithms or software that are central to the research but not yet described in published literature, software must be made available to editors and reviewers. We strongly encourage code deposition in a community repository (e.g. GitHub). See the Nature Portfolio guidelines for submitting code & software for further information.

## Data

Policy information about availability of data

All manuscripts must include a data availability statement. This statement should provide the following information, where applicable:

- Accession codes, unique identifiers, or web links for publicly available datasets
- A description of any restrictions on data availability
- For clinical datasets or third party data, please ensure that the statement adheres to our policy

This study used publicly available protein fitness datasets for computational evaluation: ProteinGym benchmark dataset [https://doi.org/10.48550/arXiv.2205.13760, https://doi.org/10.1101/2023.12.07.570727]; the fitness datasets of high-order mutants for three proteins, including GB1 [https://doi.org/10.7554/eLife.16965], CreiLOV [https://doi.org/10.1021/acssynbio.2c00662], and ParD3 [https://doi.org/10.7554/eLife.60924, https://doi.org/10.1038/s41559-022-01688-0]. The following PDB structures were used: 1PGA [https://doi.org/10.2210/pdb1PGA/pdb], 1N9L [https://doi.org/10.2210/pdb1N9L/pdb], 3CP5 [https://doi.org/10.2210/pdb3CP5/pdb], 6CUK [https://doi.org/10.2210/pdb6CUK/pdb], 6CUN [https://doi.org/10.2210/pdb6CUN/pdb]. All experimental data are available in the main text and the Supplementary Information. Plasmids encoding Rma cytochrome c variants reported in this study are available for research purposes from Y.Y. under a material transfer agreement (MTA) with the University of California Santa Barbara.

## Research involving human participants, their data, or biological material

Policy information about studies with human participants or human data. See also policy information about sex, gender (identity/presentation), and sexual orientation and race, ethnicity and racism.

| | |
|---|---|
| Reporting on sex and gender | N/A |
| Reporting on race, ethnicity, or other socially relevant groupings | N/A |
| Population characteristics | N/A |
| Recruitment | N/A |
| Ethics oversight | N/A |

Note that full information on the approval of the study protocol must also be provided in the manuscript.

# Field-specific reporting

Please select the one below that is the best fit for your research. If you are not sure, read the appropriate sections before making your selection.

☒ Life sciences ☐ Behavioural & social sciences ☐ Ecological, evolutionary & environmental sciences

For a reference copy of the document with all sections, see nature.com/documents/nr-reporting-summary-flat.pdf

# Life sciences study design

All studies must disclose on these points even when the disclosure is negative.

| | |
|---|---|
| Sample size | All the biocatalytic reactions were run in triplicates by following the common practice of the field. |
| Data exclusions | No data was excluded, investigators were blinded to group allocation during data collection and/or analysis. |
| Replication | All the biocatalytic validation reactions were performed in triplicates and all attempts at replication were successful. |
| Randomization | Not applicable |
| Blinding | Not applicable |

# Reporting for specific materials, systems and methods

We require information from authors about some types of materials, experimental systems and methods used in many studies. Here, indicate whether each material, system or method listed is relevant to your study. If you are not sure if a list item applies to your research, read the appropriate section before selecting a response.

## Materials & experimental systems

| n/a | Involved in the study |
|---|---|
| [x] | [ ] Antibodies |
| [x] | [ ] Eukaryotic cell lines |
| [x] | [ ] Palaeontology and archaeology |
| [x] | [ ] Animals and other organisms |
| [x] | [ ] Clinical data |
| [x] | [ ] Dual use research of concern |
| [x] | [ ] Plants |

## Methods

| n/a | Involved in the study |
|---|---|
| [x] | [ ] ChIP-seq |
| [x] | [ ] Flow cytometry |
| [x] | [ ] MRI-based neuroimaging |

## Plants

| | |
|---|---|
| Seed stocks | N/A |
| Novel plant genotypes | N/A |
| Authentication | N/A |
